# Early Warning Signals in Ecological Time-Series

**DOI:** 10.3390/e28060628

**Published:** 2026-06-02

**Authors:** Roberto Alvarez-Martinez, Pedro Miramontes

**Affiliations:** 1 Laboratorio de Biología Cuantitativa y Sistemas Complejos, Facultad de Ciencias Naturales, Universidad Autónoma de Querétaro, Querétaro 76269, Mexico; 2 Departamento de Matemáticas, Facultad de Ciencias, Universidad Nacional Autónoma de México, Ciudad de México 04510, Mexico; pmv@ciencias.unam.mx; 3 Facultad de Ciencias, Universidad Autónoma de San Luis Potosí, San Luis Potosí 78295, Mexico

**Keywords:** early warning signals, critical transitions, regime shifts, critical slowing down, ecological resilience, bifurcation, tipping points, time-series analysis, autocorrelation, variance

## Abstract

Ecosystems can undergo abrupt, often irreversible transitions between alternative states—phenomena termed critical transitions or regime shifts—with profound consequences for biodiversity, ecosystem services, and human well-being. Early warning signals (EWSs) derived from time-series analysis offer the prospect of anticipating such transitions before they occur, potentially enabling preventive management intervention. This review provides a comprehensive synthesis of EWS methods for ecological systems, encompassing theoretical foundations, statistical indicators, empirical applications, and emerging methodological frontiers. We examine the dynamical basis of EWS in critical slowing down theory, wherein systems approaching bifurcation points exhibit characteristic statistical signatures including rising autocorrelation, increasing variance, and spectral reddening. We present a systematic overview of proposed indicators discuss moving-window frameworks for their computation, and critically evaluate preprocessing requirements and sensitivity to analytical choices. Empirical applications across major ecosystem types—including lakes, coral reefs, grasslands, forests, and marine fisheries—reveal both successes and limitations, with EWS performance depending critically on data quality, transition mechanism, and system-specific dynamics. We address recent advances including machine learning approaches, non-equilibrium thermodynamic indicators, multivariate extensions, and the important distinction between bifurcation-induced, noise-induced, and rate-induced tipping. We conclude with recommendations for specialists, emphasizing the integration of EWS within broader monitoring frameworks, systematic sensitivity analysis, and the interpretation of indicators as probabilistic assessments of changing resilience rather than deterministic predictions of imminent collapse.

## 1. Introduction

The anticipation of abrupt, discontinuous changes in complex systems represents one of the most consequential challenges confronting contemporary science. Across disciplines ranging from climate science and ecology to epidemiology, neuroscience, and financial economics, some dynamical systems can exhibit the capacity to undergo rapid transitions between qualitatively distinct states, phenomena variously termed critical transitions, regime shifts, or tipping points [1,2]. In ecological contexts, such transitions carry profound implications: the collapse of productive fisheries, the eutrophication of clear-water lakes, the desertification of rangelands, and the die-off of coral reefs exemplify catastrophic reorganizations that may prove difficult or impossible to reverse on management-relevant timescales [3,4,5]. The development of methods capable of anticipating these transitions before they occur has accordingly emerged as a priority at the intersection of theoretical ecology, applied mathematics, and environmental policy.

Since the dawn of civilization, humanity has devoted substantial intellectual resources to forecasting catastrophes of all kinds, encompassing natural disasters, floods, earthquakes, and eclipses, and, more recently, epidemics as well as economic and financial crises. Setting aside attempts by diviners, oracles, chiromancers, and the like, systematic stability analysis emerged from celestial mechanics (Lagrange; Laplace), and the formal notion of bifurcation was introduced by Poincaré [6] in his study of the equilibrium figures of rotating self-gravitating fluid masses. This conceptual apparatus would later become foundational for the qualitative theory of dynamical systems. A more explicitly catastrophist offshoot was developed by Thom [7] and popularized by Zeeman [8], but catastrophe theory eventually fell into disuse following severe methodological criticisms [9].

The theoretical foundation for early warning signals (EWSs) of critical transitions rests primarily on the phenomenon of critical slowing down (CSD), which originated in statistical physics. Van Hove [10] first identified it through the divergent spin correlations responsible for critical magnetic scattering near the Curie point, and Landau and Khalatnikov [11] formalized it via the relaxational dynamics of the order parameter, yielding $\tau \propto \xi^{2}$ at criticality, a result later generalized by Hohenberg and Halperin [12] into the modern theory of dynamic critical phenomena ($\tau \propto \xi^{z}$). Wissel [13] subsequently transposed CSD from continuum critical phenomena to finite-dimensional dynamical systems approaching local bifurcations: as a system approaches a fold (saddle-node) bifurcation—the most commonly invoked type in ecological applications—the dominant eigenvalue of the linearized system approaches zero, causing the characteristic return time to diverge as $\tau \propto {\left|\lambda \right|}^{-1}$. This progressive loss of recovery capacity leaves characteristic statistical signatures in time-series data: increasing temporal autocorrelation, as the system retains memory of past states for longer periods, and increasing variance, as perturbations accumulate faster than the system can dissipate them [13].

The intellectual lineage of EWS research extends to foundational work across multiple fields. In ecology, Holling’s [14] distinction between engineering resilience (return time to equilibrium) and ecological resilience (magnitude of perturbation a system can absorb) established the conceptual framework within which CSD-based indicators acquired ecological meaning. The explicit development of statistical early warning indicators for ecological regime shifts began in earnest with the seminal contributions of Scheffer et al. [1] and Carpenter and Brock [15], who demonstrated theoretically that rising variance could herald approaching transitions in lake ecosystems. Dakos et al. [16] subsequently showed that increasing autocorrelation preceded major climate transitions in paleoclimate records, catalyzing a substantial research effort that has sought to validate, refine, and extend these methods across diverse systems and scales.

Despite the elegance of the underlying theory and the accumulation of supportive evidence from experimental, paleoclimate, and modeling studies, the translation of EWS from theoretical promise to operational forecasting tool remains incomplete. Several fundamental challenges constrain the practical utility of CSD-based indicators. First, the statistical power to detect early warning signals depends critically on data quality, time-series length, sampling frequency, measurement error, and the magnitude of natural variability all influence detection probability [17,18]. Many ecological monitoring programs yield data of insufficient length or resolution for reliable EWS detection, and even high-quality records may produce ambiguous signals [19]. Second, not all ecological transitions arise through the gradual approach to bifurcation that generates classical CSD signatures. Noise-induced tipping, in which stochastic perturbations drive a system across a separatrix before the bifurcation is reached, rate-induced tipping, in which parameters change faster than the system can track, and transitions driven by acute external forcing may all occur without the characteristic slowing down that precedes bifurcation-induced transitions [20,21,22]. Third, even when detectable EWSs precede a transition, they provide information only about the approach to instability, not about the precise timing, magnitude, or nature of the impending change [23,24]. The gap between detecting “something is changing” and predicting “what will happen and when” remains substantial.

Recent years have witnessed significant methodological advances that expand the EWS toolkit beyond its original foundations. Information-theoretic approaches, including Fisher information [25,26] and various entropy measures [27], offer alternative perspectives on system organization and stability. Multivariate extensions leverage covariance structure and eigenvalue dynamics to detect resilience loss in high-dimensional ecological communities [28,29]. Machine learning approaches, exemplified by deep neural networks trained on simulated tipping scenarios, can detect nonlinear precursors that escape low-order summary statistics [30,31]. Dynamic network biomarkers identify emergent modules of tightly correlated variables whose collective behavior diverges from the rest of the system prior to transition [32,33]. Non-equilibrium thermodynamic indicators derived from landscape-flux theory may provide earlier warnings than classical CSD-based metrics [34]. Spatial EWSs complement temporal analysis, particularly in patterned ecosystems where changes in vegetation structure may presage collapse [35,36]. These developments collectively represent what may be termed a second generation of EWS methods—approaches that are multi-source, probabilistic, and explicitly designed to accommodate nonstationarity and observational limitations.

Several comprehensive reviews have addressed the EWS literature in recent years, and the present work builds upon, yet is distinctly positioned relative to, these prior syntheses. Scheffer et al. [37] and Dakos et al. [38] established the methodological foundations and empirical validation framework for CSD-based indicators; Clements & Ozgul [39] synthesized biological indicators of transitions; George, Kachhara, & Ambika [40] provided a topical review of detection methods spanning conventional CSD indicators, recurrence-based measures, and machine learning approaches for complex systems broadly; and, most recently, Dakos et al. [41] provided a cross-system audit of where, how, and which early warnings have been applied across climate, ecological, and human systems over the past two decades. These syntheses are broad by design, spanning Earth system tipping elements, socioeconomic systems, and biomedical applications.

The present review complements this body of work by pursuing three emphases that remain neglected in the existing literature. First, it restricts scope to ecological systems—lakes, coral reefs, grasslands, forests, and marine environments—enabling a mechanistically grounded, ecosystem-specific treatment of each transition type. Second, it integrates explicit methodological guidance on the analytical pipeline: windowing strategies, detrending and de-seasonalizing procedures, trend detection via Kendall’s τ, and sensitivity analysis protocols (Section 3)—guidance essential for practitioners seeking to implement EWSs in monitoring programs but underrepresented in narrative syntheses. Third, by anchoring each ecosystem section in the tipping-mechanism typology of Section 2.6, this review provides a principled framework for evaluating when CSD-based indicators are theoretically expected to perform—and when they are not—across mechanistically heterogeneous systems. Together, these emphases aim to provide ecological researchers and managers with a consolidated, method-focused reference grounded in ecosystem dynamics.

The present provides a comprehensive synthesis of early warning signal methods for critical transitions in ecological systems, with particular emphasis on time-series approaches. We pursue several objectives. First, we establish the theoretical foundations linking dynamical systems theory, bifurcation analysis, and the statistical signatures of critical slowing down (Section 2). Second, we present a systematic overview of proposed EWS indicators, including both classical CSD-based metrics and more recent developments, with attention to their mathematical formulations, data requirements, and known limitations (Section 3). Third, we review empirical applications across major ecosystem types: lakes, coral reefs, grasslands, forests, and marine systems—evaluating the conditions under which EWSs have succeeded, failed, or produced ambiguous results (Section 4). Fourth, we discuss emerging methodological frontiers, including machine learning integration, multivariate extensions, and the critical challenge of prospective validation (Section 5). Finally, we offer recommendations for researchers and practitioners seeking to apply EWS in monitoring and management contexts, emphasizing the importance of methodological transparency, appropriate null model construction, and integration with mechanistic understanding.

Throughout, we maintain a perspective of informed pragmatism. Early warning signals represent neither infallible oracles nor theoretical curiosities without practical value, but rather tools whose utility depends critically on appropriate application, transparent reporting of uncertainties, and integration within broader frameworks of ecological understanding and adaptive management. As Anthropocene pressures accelerate and the prospect of crossing planetary boundaries becomes increasingly tangible [42], the capacity to anticipate critical transitions, even imperfectly, assumes growing urgency. Time-series-based early warning methods, continuously refined and thoughtfully applied, offer one of the most promising pathways toward this anticipatory capacity.

## 2. Theoretical Foundations of Early Warning Signals in Ecology

### 2.1. Critical Transitions, Resilience, and Tipping Points

A fundamental insight from dynamical systems theory is that many complex systems, including ecosystems, can exhibit multiple stable states under identical external conditions: a phenomenon termed multistability or alternative stable states [1,43]. Classic examples pervade ecology: shallow lakes may persist in either a clear-water state dominated by submerged macrophytes or a turbid state dominated by phytoplankton [44,45]; semi-arid rangelands can maintain either productive grassland or degraded bare-soil configurations [5,46]; coral reefs may exist as coral-dominated or macroalgae-dominated communities [4,47]; and forests can occupy either closed-canopy or open savanna states [48,49]. The existence of alternative stable states implies that ecosystem dynamics are governed not merely by external environmental forcing, but critically by internal feedback mechanisms that can maintain, or destabilize, particular configurations.

A tipping point (also termed critical threshold or bifurcation point) represents a critical value of an environmental driver or system parameter at which the current stable state loses its stability, precipitating an abrupt transition to an alternative state [2,37]. When such thresholds are crossed—whether through gradual environmental change (e.g., increasing nutrient loading; rising temperature) or acute perturbation (e.g., extreme drought; overharvesting)—the ecosystem may undergo rapid, discontinuous, and often unexpected reorganization [1,3]. These abrupt transitions, variously termed critical transitions, regime shifts, or catastrophic shifts in the ecological literature, are distinguished from smooth, continuous responses to environmental change by their nonlinear character and the disproportionate magnitude of ecosystem response relative to the incremental change in the driving variable [37,50].

From the perspective of dynamical systems theory, critical transitions in ecosystems are typically associated with local bifurcations, i.e., qualitative changes in the topological structure of a system’s phase space that occur as parameters vary continuously [51,52]. The most commonly invoked bifurcation types in ecological contexts are the fold (saddle-node) bifurcation and the cusp catastrophe, both of which can generate the characteristic phenomenology of critical transitions: abrupt state changes, bimodality in state distributions, and hysteresis [1,53]. In a fold bifurcation, a stable equilibrium and an unstable equilibrium collide and annihilate as a control parameter crosses a critical threshold, leaving the system with no local attractor and forcing a rapid transition to a distant alternative state [51]. The cusp catastrophe extends this framework to two-parameter systems, yielding a bifurcation surface that predicts regions of bistability, critical thresholds, and the possibility of hysteresis loops [7,53].

Hysteresis, the dependence of the system’s state not only on current conditions but also on its history, constitutes a particularly consequential feature of fold-type bifurcations in ecosystems [1,43]. When an ecosystem crosses a tipping point and transitions to an alternative state, returning to the original state typically requires reversing the driving parameter well beyond the original threshold value, often to substantially lower (or higher) levels [37]. This asymmetry between forward and backward transitions implies that regime shifts may be difficult or effectively impossible to reverse on management-relevant timescales, even if the original stressor is removed [3,54]. The potential for such irreversibility underscores the critical importance of anticipating approaching tipping points before they are crossed.

### 2.2. Ecological Resilience: Definitions and Quantification

The concept of ecological resilience provides the theoretical foundation for understanding how ecosystems respond to perturbations and, critically, for anticipating when they may be approaching critical transitions. Holling’s seminal distinction between “engineering resilience” (the rate of return to equilibrium following perturbation) and “ecological resilience” (the magnitude of perturbation a system can absorb before transitioning to an alternative state) established the conceptual framework that continues to guide resilience research [14,55]. In systems exhibiting alternative stable states, ecological resilience can be conceptualized geometrically as the size, depth, or width of the basin of attraction surrounding the current equilibrium in state space [1,56,57].

Mathematically, resilience near equilibrium is often quantified through the dominant eigenvalue of the system’s Jacobian matrix evaluated at the equilibrium point [58]. For a system at a stable equilibrium, the dominant eigenvalue $\lambda_{1}$ is negative, and its magnitude $|\lambda_{1}|$ determines the rate at which small perturbations decay—larger magnitudes indicate faster recovery and hence greater local stability [38,59]. The reciprocal of $|\lambda_{1}|$ provides an estimate of the characteristic return time τ to equilibrium:

(1) $$\tau =\frac{1}{|\lambda_{1}|}$$

As a system approaches a fold bifurcation, the dominant eigenvalue approaches zero from below, causing the characteristic return time to diverge—a phenomenon termed as critical slowing down [13,37,60]. This progressive lengthening of recovery times constitutes the fundamental dynamical basis for most early warning signals of approaching critical transitions.

Beyond local stability metrics, alternative approaches to quantifying resilience include measures based on basin geometry (basin width, depth, or volume), potential energy landscapes, stochastic stability (mean first-passage times between basins), and information-theoretic quantities [61,62,63]. Each approach captures different aspects of system robustness and may exhibit different sensitivities to approaching transitions. Integrating multiple resilience metrics may therefore provide more robust early warning than any single indicator [39,64].

### 2.3. Critical Slowing Down: The Dynamical Basis of Early Warning Signals

Critical slowing down (CSD) refers to the phenomenon whereby a dynamical system’s rate of recovery from perturbations decreases as it approaches a bifurcation point [13,60]. This slowing of internal dynamics constitutes the theoretical cornerstone upon which most statistical early warning signals are constructed [16,37]. The phenomenon arises generically near fold bifurcations because the linearized dynamics around equilibrium become progressively weaker as the equilibrium approaches the bifurcation point—the “restoring force” that returns the system to equilibrium diminishes, yielding increasingly sluggish dynamics [51,65].

For ecological systems subject to continuous stochastic forcing (environmental variability; demographic stochasticity), critical slowing down produces characteristic statistical signatures in time-series data that can serve as early warning signals [15,16]. The three most widely studied indicators are increasing standard deviation, increasing temporal autocorrelation, and increasing variance.

The mapping between CSD and catastrophic ecological transitions is, however, subtler than the bifurcation analogy alone suggests. Classical CSD in statistical physics is a property of second-order (continuous) phase transitions, where the correlation length diverges; the catastrophic regime shifts of ecological interest, by contrast, are more closely analogous to first-order transitions, with discontinuous state changes and hysteresis. Within the dynamical-systems framework, the fold bifurcation plays the role not of the critical point itself but of the spinodal, the limit of linear stability of the metastable branch, at which the order-parameter relaxation time diverges, recovering CSD in this restricted sense [66]. The empirical reliability of CSD-based indicators has also been the subject of substantial debate: Kéfi et al. [67] show that EWSs can also precede non-catastrophic transitions and therefore do not, by themselves, discriminate transition type; Drake [68] and Boettiger, Ross, and Hastings [24] explicitly map the regimes in which CSD-based indicators are and are not expected to apply. The signatures discussed below should therefore be understood as necessary but not sufficient conditions for an approaching transition; the detailed applicability conditions, including noise-, rate-, and shock-induced tipping mechanisms in which CSD does not arise, are developed in Section 2.5 and Section 2.6.

A further complication, recognized in the recent theoretical literature, is that the qualitative direction of the CSD signal is itself model-dependent. Two distinct mechanisms have been shown to produce critical speeding up (CSU)—a progressive decrease of the characteristic return time, and a corresponding fall in variance and autocorrelation, as the transition is approached—in direct contrast to classical CSD. Pomeau and Le Berre [69], in the setting of stick-slip relaxation oscillations, showed that when the slow manifold of a system develops a finite-time singularity, the response to external noise speeds up rather than slows down before the transition; an experimental realization of this mechanism is provided in [70]. In an ecological setting, Titus and Watson [71] demonstrated that when a slow parameter change compresses the basin of attraction—rather than flattening the potential, as in the canonical fold scenario underlying CSD—the potential well steepens and the stationary variance and lag-1 autocorrelation decrease as the transition is approached. Both mechanisms are theoretically robust, and at least one (the Titus–Watson Allee-effect mechanism) is directly relevant to ecological systems for which CSD-based indicators have been routinely deployed.

The practical implication is that the sign of the expected trend in variance and autocorrelation is not universal: depending on the geometry of the parameter change and the structure of the underlying potential, EWSs may manifest as rising or falling indicators. This complication has two consequences for EWS deployment that are developed further in Section 2.5 and Section 2.6. First, the applicability of CSD-based methods presupposes not only that the system is approaching a bifurcation, but that the bifurcation is of the type for which the potential flattens rather than narrows. Second, standard one-sided trend tests (Kendall’s τ as commonly applied; see Section 3.1.3) are not sufficient on their own when the sign of the expected trend is unknown a priori. Either two-sided tests, or sign predictions derived from a mechanistic model of the focal system, are required. In what follows, we present the indicator signatures expected under classical CSD; readers should bear in mind that the magnitude of trend changes, rather than its sign alone, is often the more reliable diagnostic when the underlying mechanism is uncertain.

A comprehensive review of EWS metrics can be found in the Section 3.

### 2.4. Additional Statistical Indicators

Beyond autocorrelation and variance, several additional statistical indicators have been proposed based on the expected dynamical changes near bifurcations. These include (among many others) the following:

Skewness: As the system approaches a tipping point, the state distribution may become increasingly asymmetric due to the nonlinear shape of the stability landscape, yielding rising skewness prior to transition [72]. Flickering and bimodality: In bistable systems near critical thresholds, stochastic fluctuations may occasionally drive the system across the separatrix into the alternative basin of attraction before returning, producing sporadic excursions visible as “flickering” between states [73,74]. As the potential barrier between attractors erodes, the frequency distribution of the state variable may become detectably bimodal, with persistence in alternating states and elevated frequency of extreme excursions constituting operational warning metrics [74,75,76,77]. Spectral reddening: Critical slowing down shifts the power spectrum of fluctuations toward lower frequencies (longer wavelengths), a phenomenon detectable as increasing spectral density at low frequencies [16,78,79]. Conditional heteroscedasticity: Increased sensitivity to perturbations near tipping points may manifest as greater variability in variance itself, detectable through ARCH/GARCH-type analyses [80]. Spatial indicators: In spatially extended systems, critical slowing down can manifest as increased spatial correlation, increased spatial variance, and changes in spatial pattern structure (e.g., patch-size distributions; connectivity) [5,35,36]. These spatial analogs of temporal EWSs are particularly valuable when long time series are unavailable, offering resilience diagnostics from landscape snapshots alone.

These indicators are not uniformly informative across all transition mechanisms, and their diagnostic value depends critically on the class of tipping generating the transition—a distinction developed formally in Section 2.5. Flickering and bimodality are specifically associated with bistable systems approaching fold bifurcations (B-tipping): they require the coexistence of two basins of attraction separated by a finite potential barrier, a structural condition absent under N-tipping (where stochastic escape can produce superficially similar excursions without genuine bistability), R-tipping, and S-tipping [73,81]. Spectral reddening and conditional heteroscedasticity may manifest under both B-tipping and externally forced dynamics, since increasing environmental variability can independently redden spectra and inflate volatility without any approach to a bifurcation point [78,80]. Spatial indicators share the B-tipping specificity of their temporal counterparts, but offer an additional advantage: because they aggregate information across spatially replicated observations, they may detect collective slowing down even when individual time series are too short for reliable univariate EWS detection [35,36]. This heterogeneity in mechanism-specificity—wherein some indicators are theoretically grounded only for B-tipping while others can arise spuriously under alternative mechanisms—motivates the explicit tipping typology introduced in Section 2.5, which provides the principled basis for selecting and interpreting appropriate indicator subsets for a given system. It also reinforces the caution against the standard one-sided sign assumption discussed in Section 2.3: both the direction and the very presence of an EWS signal are mechanism-dependent.

### 2.5. Scope, Limitations, and Applicability of CSD-Based Early Warning Signals

EWSs grounded in critical slowing down occupy a specific and well-defined niche within the broader landscape of transition forecasting: they are designed to detect the fingerprints of bifurcation-driven (B-tipping) transitions, in which a slowly changing parameter gradually erodes the stability of a fixed point until the system loses it entirely. This specificity is a strength—it connects EWSs directly to rigorous dynamical systems theory—but it is also a constraint, because ecological transitions can be generated by at least three other mechanistically distinct tipping types, each with its own relationship to CSD-based indicators (see Section 2.6).

Despite compelling theoretical foundations and mounting empirical support, the application of CSD-based EWSs is subject to four important limitations that constrain their reliability for prospective prediction of ecosystem transitions  [22,23,63]:

1. Detection power is data-limited. The statistical power to detect early warning signals depends critically on time-series length, sampling frequency, measurement error, and the magnitude of natural variability [17,23]. Crucially, sampling frequency must be interpreted in the context of time-scale separation: the sampling interval must be sufficiently short to resolve the intrinsic fast dynamics of the system and fluctuations and recovery trajectories from small perturbations, while the external forcing parameter driving the system toward the bifurcation operates on a much slower timescale [37,38]. This separation of timescales is a core requirement of the CSD framework; when it is violated, either because sampling is too coarse to capture internal fluctuations, or because external forcing changes too rapidly relative to the system’s own dynamics, the statistical precursors of fold bifurcations may not accumulate detectably, or may be confounded with non-stationarity artifacts [82,83]. In practice, this means that the appropriate sampling frequency is system-specific and must be calibrated against the characteristic return time of the focal state variable. Short or noisy time series may yield high false-positive or false-negative rates, limiting operational predictive capacity [18,38].
2. EWSs are transition-mechanism specific. Not all ecological transitions are preceded by detectable critical slowing down. Transitions driven primarily by external forcing (e.g., abrupt climate shifts; acute disturbances) rather than gradual erosion of resilience may occur without the slow approach to instability that generates classical EWSs [82,84]. Transitions arising from noise-induced, rate-induced, or shock-induced tipping (N-, R-, and S-tipping; see Section 2.6) do not produce the characteristic statistical precursors of fold bifurcations [20,21].
3. EWS signal instability, not timing. Even when EWSs are detectable, they provide information only about the approach to instability, not about the precise timing, magnitude, or nature of the impending transition [24,37]. Translating generic indicators into specific, actionable predictions remains a substantial challenge.
4. High-dimensional complexity obscures signals. Regime shifts in real ecosystems often involve complex, high-dimensional dynamics, spatial heterogeneity, multiple interacting stressors, and cascading effects across trophic levels—complexities that may obscure, amplify, or fundamentally alter the expression of critical slowing down [85,86].

These limitations underscore the importance of interpreting EWSs as probabilistic indicators of changing resilience rather than deterministic predictions of imminent collapse and of integrating statistical indicators with mechanistic understanding, experimental validation, and adaptive management frameworks  [39,63,64].

Table 1 summarizes the set of indicators used in this study, together with their mathematical definitions, the statistical property each one measures, the change expected as the system approaches a transition, and the main limitations or caveats associated with their use.

### 2.6. A Typology of Tipping Mechanisms and Their EWS Signatures

**Figure 1:**
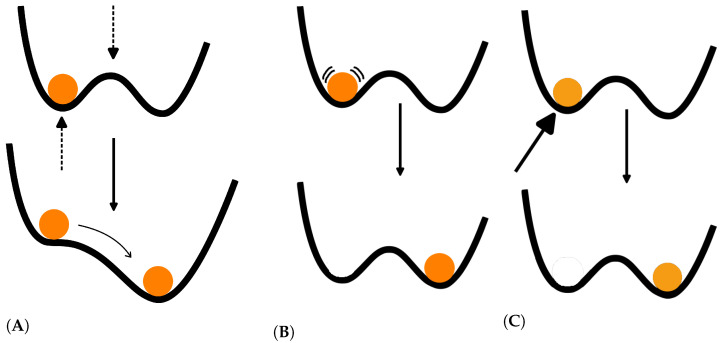
Three mechanisms that provoke sudden, irreversible changes in a system (see text). (**A**) Bifurcation-induced tipping: a change in a system parameter causes one local minimum to disappear. (**B**) Noise-induced tipping: thermal fluctuations may reach a threshold that allows the state to jump over the barrier. (**C**) Shock-induced tipping: an external perturbation (arrow) may be large enough to allow the system state to overcome the barrier.

The utility and reliability of any given early warning indicator depends fundamentally on the mechanism by which the transition is generated. Following Ashwin et al. [95] and Lenton et al. [83], four mechanistically distinct classes of tipping can be identified for a general stochastic dynamical system $\dot{x}=f\left(x;\;\mu \right)+\sigma \xi \left(t\right)$, where μ is a slowly evolving control parameter, σ is noise amplitude, and ξ(t) is a stochastic forcing term. Identifying which class applies to a given system is a prerequisite for responsible EWS deployment. See Figure 1.

#### 2.6.1. B-Tipping (Bifurcation-Induced)

The deterministic skeleton $\dot{x}=f\left(x;\;\mu \right)$ undergoes a bifurcation as μ approaches a critical value $\mu_{c}$. The most common case in ecology is the fold (saddle-node) bifurcation, in which two fixed points—one stable, one unstable—collide and annihilate as $\mu \to \mu_{c}$. The dominant eigenvalue $\lambda_{1}\left(\mu \right)$ of the Jacobian $Df(x^{*};\mu )$ at the stable fixed point $x^{*}$ satisfies $\lambda_{1}\to 0^{-}$ as $\mu \to \mu_{c}$ (Equation (1)). This eigenvalue convergence is the mechanism of CSD: the characteristic return time $\tau_{r}=-1/\lambda_{1}\to \infty$, so perturbations decay increasingly slowly, inflating variance and autocorrelation in the observed process. CSD is a necessary consequence of B-tipping in systems well-described by a fold bifurcation, making it in principle detectable from time-series data  [51,52]. Other bifurcation types (Hopf, transcritical, and pitchfork) can also generate CSD, though the precise indicator dynamics differ: a Hopf bifurcation produces a complex conjugate pair of eigenvalues whose real part approaches zero, generating increasing oscillatory variance rather than a monotonic AR(1) rise [73].

#### 2.6.2. N-Tipping (Noise-Induced)

The deterministic skeleton retains its stable fixed point ($\lambda_{1}0$ throughout), but sufficiently large stochastic fluctuations can kick the system over the basin boundary into an alternative attractor  [81]. Formally, the probability of an N-tipping event in time *T* follows a Kramers-type escape rate $P_{\mathrm{esc}}\approx 1-\exp\left(-T/\tau_{K}\right)$, where $\tau_{K}\propto \exp\left(\Delta V/\sigma^{2}\right)$, and ΔV is the depth of the potential well. Because $\lambda_{1}$ remains negative and bounded away from zero, CSD indicators do not rise systematically before N-tipping events. Indeed, an increase in σ that raises escape probability will inflate variance in a way that superficially resembles a CSD signal, generating false positives [20]. Distinguishing N-tipping from B-tipping based on time-series data alone is a fundamental inferential challenge [81,82].

A particularly insidious practical complication arises when the noise amplitude σ itself increases over time—for instance, due to rising environmental variability under anthropogenic forcing. In such cases, the stationary variance of the observed process (Equation (3)) will inflate even though the dominant eigenvalue $\lambda_{1}$ remains bounded away from zero and no bifurcation is being approached. The resulting trend in variance is statistically indistinguishable from a genuine CSD signal, generating false positives that could trigger unwarranted management interventions. This σ-driven variance inflation is not merely a theoretical concern: increasing climate variability in precipitation, temperature, and disturbance regimes means that many ecological monitoring programs are likely operating under non-stationary noise conditions. Researchers should therefore complement variance-based indicators with return-rate estimates (e.g., from pulse perturbation experiments or drift-diffusion fitting), which are more directly tied to $\lambda_{1}$ and less sensitive to changes in forcing amplitude [60,96]. When experimental perturbations are not feasible, comparing variance trends against independent proxies of external forcing variability can help distinguish genuine resilience loss from noise-driven inflation.

When experimental perturbations are not feasible, comparing variance trends against independent proxies of external forcing variability can help distinguish genuine resilience loss from noise-driven inflation. A related and often overlooked source of spurious variance trends arises from transitions between measurement instruments: changes in sensor uncertainty alter the effective signal-to-noise ratio of the observed time series, introducing biases in variance and lag-1 autocorrelation that can be misattributed to genuine shifts in system resilience [97].

#### 2.6.3. R-Tipping (Rate-Induced)

A stable state can be lost if the control parameter μ(t) changes faster than the system can track it, even if the system would remain stable under quasi-static conditions at every instantaneous value of μ [15,21]. Formally, R-tipping occurs when the rate $|\dot{\mu }|$ exceeds a critical threshold that depends on the geometry of the co-dimension-1 stable manifold in the (μ,x) phase plane. Because $\lambda_{1}$ never approaches zero—the system is always formally stable relative to its current μ value—no CSD signal is generated before R-tipping. Temporal autocorrelation and variance may in fact remain approximately constant or even decrease slightly as the system tracks a moving attractor under rapid forcing. R-tipping is particularly relevant for ecological systems subject to rapidly intensifying anthropogenic pressures [15].

#### 2.6.4. S-Tipping (Shock-Induced)

An abrupt, large-amplitude perturbation, independent of the system’s proximity to a bifurcation, displaces the state variable directly into an alternative basin of attraction [81]. Examples include catastrophic coral bleaching following a single anomalous thermal event or forest loss following a megafire. By definition, no precursory dynamics precede the event, and  no CSD signal is expected or observed. Post-shock behavior (recovery rate; trajectory in state space) may yield retrospective information about resilience but cannot provide prospective warning [98,99].

## 3. Time Series Early Warning Methods

Several time-series measures have been proposed as early warning indicators for ecological systems. Typically, the strategy involves calculating these metrics on ecological time-series data, such as population abundance, biomass, and vegetation cover over time, and monitoring their trends. A systematic increase in any of these indicators could indicate that that the system approaches a critical threshold [38]. In this section, we summarize the most studied indicators, their theoretical foundations, and the expected behavior as the system approaches critical transitions (see Table 1).

**Figure 2:**
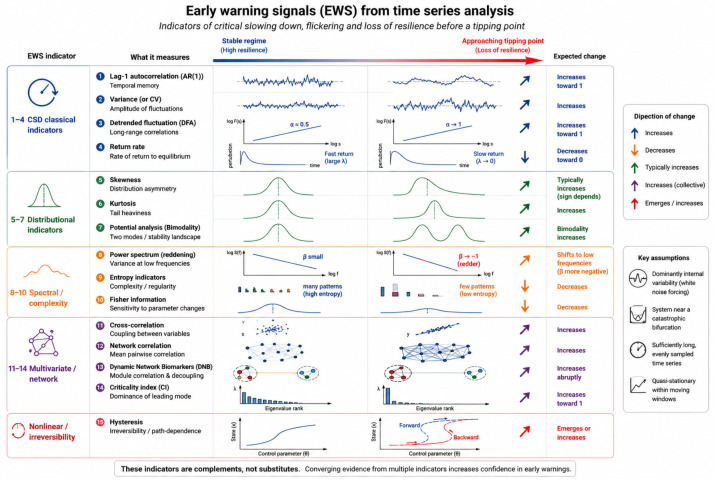
Early warning signals (EWSs) from time-series analysis: a graphical synthesis. Overview of the fifteen indicators detailed in Table 1, organized into five functional groups. Groups 1–4: Classical CSD indicators —lag-1 autocorrelation (AR(1)), variance (or CV), detrended fluctuation analysis (DFA), and return rate—directly reflect the lengthening recovery time as the dominant eigenvalue approaches zero near a fold bifurcation. Groups 5–7: Distributional indicators—skewness, kurtosis, and potential-analysis bimodality—capture the asymmetric and bimodal deformations of the state distribution as the potential well flattens. Groups 8–10: Spectral and complexity measures—power spectrum reddening, entropy indicators, and Fisher information—track the shift of fluctuation energy toward low frequencies and the concomitant loss of dynamical complexity. Groups 11–14: Multivariate and network-based indicators—cross-correlation, network correlation, dynamic network biomarkers (DNBs), and the criticality index—exploit the increasing coherence among system components as all variables slow down collectively. Group 15: Hysteresis—as a nonlinear irreversibility indicator—detects path-dependence confirming the presence of alternative stable states. For each indicator the left panel depicts behavior under high resilience (stable regime) and the right panel under loss of resilience approaching a tipping point; the Expected change column summaries the direction of the anticipated trend (color-coded legend, inset). These indicators are complements, not substitutes: converging evidence from multiple metrics increases inferential confidence (Section 3.2).

Figure 2 provides a graphical synthesis of these indicators, grouping the fifteen metrics of Table 1 into five functional families and contrasting their typical behavior under high resilience with that on the approach to a tipping point.

### 3.1. Moving-Window Frameworks for EWS Detection

Early warning indicators derived from critical slowing down are most commonly computed within a moving-window framework, in which a statistical metric is repeatedly re-estimated on successive segments of the time series and its temporal trend is then quantified [37,38]. This approach transforms a univariate time series into a derived indicator time series, whose trajectory can reveal progressive changes in system dynamics as a critical transition is approached. The choice of window design fundamentally shapes both the sensitivity and reliability of early warning detection.

#### 3.1.1. Rolling vs. Expanding Window Approaches

Two primary windowing strategies dominate the early warning literature, each with distinct statistical properties and ecological interpretations. In the rolling (sliding) windows approach, one fixes a window length *w* (typically expressed in time units or number of observations) and slides it forward, often with substantial overlap (e.g., advancing by a single time step), so that at each time index *t* the indicator is computed from the local segment $\left\{x_{t-w+1},\ldots ,x_{t}\right\}$. This design mimics prospective real-time monitoring because each estimate uses only the most recent *w* observations, discarding older data that may reflect different dynamical regimes [38]. The resulting time series of indicator values can then be tested for systematic trends (increases or decreases) that would be consistent with approaching a bifurcation.

Rolling windows offer high sensitivity to local changes in system dynamics, making them particularly effective at detecting late-stage acceleration in early warning metrics near a transition point [83]. However, this sensitivity comes at a cost: when *w* is small (e.g., w<50 observations), variance in indicator estimates can be substantial, leading to noisy trajectories that obscure genuine trends and increase false-positive rates [18,38]. The choice of window size thus involves a fundamental trade-off between temporal resolution and estimation precision. Empirical guidelines suggest that *w* should be large enough to reliably estimate the desired statistic (e.g., at least 30–50 observations for variance or autocorrelation estimates) but small enough to track changes over the time scale of the hypothesized transition [38,64]. Some studies recommend setting *w* to approximately 50% of the total time-series length as a starting point, though this heuristic should be validated against the suspected transition time scale [38].

**Figure 3:**
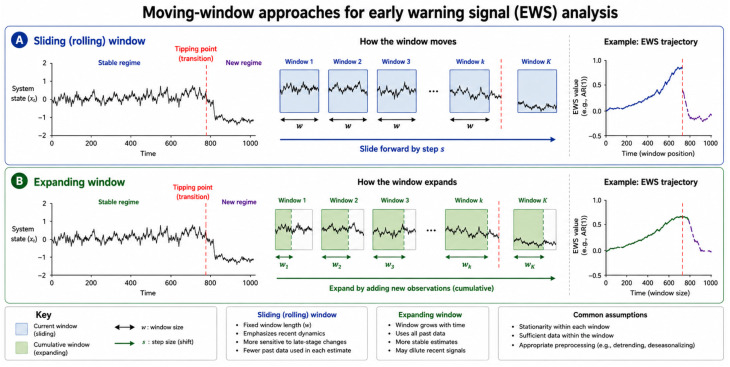
Moving-window approaches for early warning signal (EWS) analysis. (**A**) Sliding (rolling) window: a fixed-length window of width *w* advances by step *s*, retaining only the most recent *w* observations at each position. This design emphasizes local dynamics and is more sensitive to late-stage changes near a tipping point, but yields higher estimator variance when *w* is small. The right panel shows a representative EWS trajectory (e.g., AR(1)) rising as the system approaches the transition (red dashed line). (**B**) Expanding (growing) window: the window accumulates all observations from a fixed start point, increasing in size $w_{k}$ at each step. Pooling more data reduces estimator variance but can dilute recent dynamical changes, potentially masking late-stage acceleration in EWS metrics. Both approaches assume approximate stationarity within each window and require appropriate preprocessing (detrending; de-seasonalising) before indicator computation.

In contrast, the expanding (growing) windows approach incrementally increases the window size over time, computing the indicator from a cumulative segment $\left\{x_{1},\ldots ,x_{t}\right\}$ or from a fixed start point after an initial burn-in period to establish baseline conditions [38]. By pooling progressively more data, expanding windows reduce estimator variance and can provide more stable trend estimates, particularly early in the monitoring period when rolling windows would be based on limited data. However, this cumulative design has a critical limitation: if the system undergoes gradual or multi-phase changes, early observations reflecting a stable regime can dilute or mask late-stage acceleration in early warning metrics [18,83]. Expanding windows may therefore be less sensitive to recent dynamical shifts and can produce misleading trends when regime changes are not monotonic. See Figure 3.

Comparative studies on both simulated and empirical ecological time series suggest that rolling windows generally outperform expanding windows for detecting imminent transitions, particularly when the approach is gradual and window size is chosen appropriately [38,83]. Nevertheless, combining both approaches or using hybrid schemes (e.g., expanding windows with weighted observations favoring recent data) may provide complementary information for robust early warning assessment [18].

#### 3.1.2. Overlap and Computational Considerations

The degree of overlap between successive windows is another important design choice. Maximum overlap (advancing by one time step) produces the smoothest indicator trajectories and finest temporal resolution but introduces strong serial autocorrelation in the derived time series, which violates independence assumptions in many trend tests and can inflate significance estimates [38,100]. Non-overlapping windows eliminate this autocorrelation but drastically reduce the number of data points available for trend analysis, potentially missing short-lived signals. A practical compromise is to use moderate overlap (e.g., advancing by w/4 or w/2 observations), balancing temporal resolution against statistical assumptions, though the optimal choice remains context-dependent and should be evaluated via sensitivity analysis or simulation studies [18,64].

It is worth noting that serial autocorrelation in the derived indicator time series can be addressed through null model approaches, such as those proposed by [16], which generate surrogate time series that preserve the autocorrelation structure of the original data and thereby provide more robust significance estimates. Furthermore, the choice of overlap must be considered in relation to the total length of the time series *N*. The common convention of setting w=N/2 results in very few independent windows when advancing by w/2 or w/4 steps, potentially limiting the statistical power of trend detection. Practitioners should therefore consider the trade-off between window size and the number of resulting data points when designing their analysis.

#### 3.1.3. Trend Detection Methods

Once a time series of indicator values has been generated via moving windows, the next step is to assess whether it exhibits a significant temporal trend consistent with critical slowing down. The trend is most frequently quantified using Kendall’s**τ**, a rank-based, non-parametric measure of monotonic association that is robust to non-Gaussianity, outliers, and nonlinear transformations of the data [38,100]. For a derived indicator series $\left\{y_{1},\ldots ,y_{n}\right\}$, Kendall’s τ ranges from −1 (perfect negative trend) to +1 (perfect positive trend), with values near zero indicating no systematic temporal pattern. Statistical significance is typically assessed via permutation tests or asymptotic approximations, yielding a *p*-value that indicates whether the observed trend is stronger than expected under a null hypothesis of no temporal structure [38,100].

Kendall’s τ is preferred over parametric alternatives such as Pearson correlation or linear regression slopes because ecological time series often violate normality assumptions, contain outliers from extreme events, and may exhibit nonlinear trends near bifurcations [100]. However, Kendall’s τ is most powerful for detecting monotonic trends and may miss more complex patterns such as accelerating or decelerating changes [64]. Alternative approaches include fitting polynomial or exponential trend models, computing Spearman’s rank correlation (which has similar robustness properties but slightly different power characteristics), or employing change-point detection methods to identify abrupt shifts in indicator levels [18,30]. Recent work has also explored machine learning classifiers that integrate multiple indicators and their trends to improve early warning performance [30].

We further note that when the direction of the expected indicator trend cannot be predicted a priori, for example, when critical speeding up rather than slowing down may apply (Section 2.3)—the standard one-sided Kendall’s τ test should be replaced by a two-sided formulation, or the sign of the test should be informed by a mechanistic model of the focal system [71].

#### 3.1.4. Preprocessing: Detrending and De-Seasonalizing

Generic critical-slowing-down indicators such as lag-1 autocorrelation $\rho_{1}$ (AR(1) coefficient), variance $\sigma^{2}$, and related second-order statistics can be strongly biased by exogenous trends, seasonal cycles, or other forms of non-stationarity that are unrelated to proximity to a bifurcation [38]. For example, a time series with a deterministic upward trend will naturally exhibit elevated variance and autocorrelation even in the absence of critical slowing down, potentially generating spurious early warnings. Similarly, pronounced seasonality can inflate variance estimates and create periodic autocorrelation patterns that obscure genuine dynamical changes [18].

To mitigate these biases, window-based estimation is typically preceded by detrending or de-seasonalizing procedures. The most common approach applies a smoothing filter (e.g., Gaussian kernel smoothing, LOESS regression, or moving averages) to the original time series, then analyzes the residuals after subtracting the smooth trend [38]. The bandwidth of the smoothing filter should be chosen to remove low-frequency trends and seasonality while preserving the higher-frequency fluctuations that reflect critical slowing down; bandwidths that are too narrow leave spurious trends intact, while overly broad smoothing can remove genuine signals [18,38]. First-differencing (computing $\Delta x_{t}=x_{t}-x_{t-1}$) is an alternative detrending method that eliminates linear trends but can also remove or distort autocorrelation patterns, making it less suitable for early warning analysis [38].

For strongly seasonal data, preprocessing may involve fitting and removing seasonal components via classical decomposition, seasonal-trend decomposition using LOESS (STL), or more sophisticated state-space models that separate trend, seasonal, and residual components [64]. After preprocessing, early warning indicators are computed on the residual time series within each moving window, ideally isolating changes in system resilience from confounding temporal patterns. However, preprocessing choices introduce additional degrees of freedom that can influence early warning results, and sensitivity analyses should examine how conclusions depend on bandwidth selection, smoothing method, and differencing order [18,38].

### 3.2. Interpretation and Limitations of Window-Based EWSs

A significantly positive trend in lag-1 autocorrelation, variance, or related metrics computed via moving windows is commonly interpreted as consistent with reduced stability and slower recovery rates as a system approaches a critical threshold [37,83]. The theoretical foundation rests on the prediction that near a bifurcation point, the dominant eigenvalue of the linearized system approaches zero, causing perturbations to decay more slowly and fluctuations to amplify [37,65]. However, several critical limitations must be acknowledged when interpreting window-based early warnings in ecological applications.

#### 3.2.1. Specificity and Alternative Mechanisms

Increases in autocorrelation and variance are not unique to approaching bifurcations and can arise from multiple alternative mechanisms. Stochastic transitions driven by rare large perturbations (e.g., extreme weather events; invasive species arrival) can produce abrupt regime shifts without gradual loss of resilience, yet retrospective analysis may still reveal apparent early warning trends if the data are conditioned on the transition occurring [23,75]. Similarly, directional environmental change that drives the system toward a threshold can generate trends in indicators even if the transition itself is discontinuous or driven by external forcing rather than endogenous destabilization [63]. Changes in environmental variability (e.g., increasing climate extremes) can directly inflate variance estimates independently of system stability [18].

#### 3.2.2. Conditional Sampling and Retrospective Bias

Many early warning studies analyze historical time series selected specifically because they ended in a known transition, introducing conditional sampling bias that can spuriously elevate indicator trends [23,75]. If only systems that transitioned are analyzed, one may observe rising indicators simply because fluctuations happened to be large near the transition (potentially contributing to it), rather than because of systematic slowing down. Prospective monitoring of systems that do not transition is essential to assess false-positive rates and calibrate expectations [18,64].

#### 3.2.3. Window Size Sensitivity and Reporting Standards

The sensitivity of early warning detection depends critically on window size, overlap, preprocessing choices, and trend-testing methods, yet many studies report results for a single parameter combination without exploring robustness [18]. Best practices include conducting sensitivity analyses across a range of window sizes, reporting both positive and negative results, comparing multiple indicators, and validating findings against surrogate or null models that preserve key statistical properties of the data while removing hypothesized early warning signals (e.g., phase-randomized surrogates that maintain spectral properties but scramble temporal structure) [18,38,75]. Transparent reporting of methodological choices, null hypothesis specifications, and significance thresholds is essential for reproducibility and to avoid selective reporting of positive results [64].

#### 3.2.4. Practical Recommendations

Given these limitations, window-based early warnings should be interpreted cautiously and embedded within broader monitoring frameworks that integrate mechanistic understanding, multiple lines of evidence, and explicit consideration of alternative hypotheses [18,63]. Combining multiple complementary indicators (e.g., autocorrelation, variance, skewness, and spatial metrics), assessing their consistency, and contextualizing trends within ecological theory can strengthen inference [30,37,64]. Where possible, experimental manipulations or comparative studies across replicate systems can provide stronger causal evidence than relying solely on observational time series [18]. Ultimately, early warning signals are most useful when viewed as hypothesis-generating tools that warrant further investigation rather than as definitive predictors of imminent transitions.

Taken together, the limitations reviewed in Section 3.2.1, Section 3.2.2 and Section 3.2.3 establish that CSD-based indicators computed in moving windows are necessary but not sufficient components of a rigorous early warning framework. Their statistical power is constrained by data quality and series length; their specificity is undermined by the alternative mechanisms catalogued in Section 2.6, each of which can generate statistically identical signatures without genuine proximity to a bifurcation; and their outputs remain continuous and uncertain in ways that resist direct translation into management decisions. Furthermore, as established in Section 2.3, neither the presence nor the direction of the expected indicator trend is universal: critical speeding up under certain potential geometries and mechanism-specific false positives under N-, R-, and S-tipping mean that the interpretation of any single indicator trend requires prior mechanistic context. These constraints motivate two classes of complementary approaches reviewed in Section 3.3: model-based and mechanistic methods that embed indicator computation within explicit dynamical frameworks and machine learning approaches capable of extracting nonlinear precursors that escape low-order summary statistics.

### 3.3. Complementary Models and Approaches to Detect EWSs

#### 3.3.1. Methods Based on System Dynamics (Mechanistic Models)

One way to anticipate transitions is to employ explicit dynamic models of the system and analyze its stability in real time. Unlike purely empirical approaches (metrics described above), these methods attempt to infer dynamic parameters or fit simplified models to the data, thereby obtaining indicators more directly related to bifurcation theory. Some examples include the following:

- ARIMA/AR(ρ) models with variable parameters: Fit an auto-regressive model to the series where the coefficients can change over time. Importantly, the integrated (I) component of ARIMA models also provides a principled approach to detrending the time series via differencing, removing non-stationarities prior to fitting. For example, an AR(1) model $X_{t+1}=a_{t}X_{t}+b_{t}+\varepsilon_{t}$ allows the coefficient $a_{t}$ to be estimated in each window, with an approach to 1 indicating a critical slowdown (very long return time) [38]. Extensions include threshold AR models, where different regimes are applied at different ranges of the variable.
- Potential analysis (dynamic potential): This is a non-parametric approach that reconstructs the effective potential function of the system from the data distribution or a drift-diffusion estimate. The idea is to identify changes in the stability topography: for example, the appearance of a shallow or secondary minimum in the potential may signal a decrease in resilience. Tools such as potential analysis [89] detect multiple potential wells (indicating incipient alternative states) over time.
- Nonparametric drift–diffusion estimation. This approach consists of inferring the drift term f(x) and the diffusion term g(x) directly from the time series, assuming that the underlying dynamics follow a stochastic differential equation of the form
  $$\frac{dx}{dt}=f\left(x\right)+g\left(x\right)\;\xi \left(t\right),$$
  where ξ(t) represents a stochastic noise term. As the system approaches a critical transition, the derivative of the drift $f^{\prime }\left(x\right)$—which is related to the dominant eigenvalue of the system—tends toward zero. Simultaneously, changes in the diffusion component may indicate emerging instabilities.
  Carpenter and Brock [74] developed a drift–diffusion–jump (DDJ) framework that uses nonparametric estimation to distinguish between signals driven by critical slowing down and those caused by flickering (noise-induced transitions) in ecological time series; their approach was further developed in [76] and implemented in the earlywarnings R package by Dakos et al. [38]. Although powerful, these methods require relatively long time-series data and rely on assumptions regarding the functional form of the system’s dynamics.
- Controlled experimental disturbances: In systems that allow it (e.g., an experimental lake or a greenhouse pasture), the recovery rate can be measured directly by applying minor disturbances and observing the response. A decrease in the observed return rate is the most direct signal of proximity to a tipping point. For example, Veraart et al. [96] measured, in an aquatic microcosm, that population recovery after minor disturbances became increasingly slower as the transition was approached, demonstrating a loss of resilience.

In general, model-based approaches allow the integration of known theoretical information (equations; mechanisms) and offer more robust hypothesis testing (e.g., testing whether the data agree with a bifurcation model). They also facilitate significance testing through simulation (bootstrap of fitted models). However, they depend on the quality of the model fit and can fail if the real system does not conform to the model assumptions.

#### 3.3.2. Machine Learning Approaches

With the rise of data science, machine learning techniques have been introduced to detect subtle early warning patterns. One advantage is that machine learning (ML) algorithms can combine multiple indicators or extract non-obvious features from time series, potentially improving prediction accuracy. Some recent examples include the following:

- Classifiers trained on simulated data: Large sets of synthetic time series with and without critical transitions can be generated (using simulated ecological models under various conditions), and a classifier (e.g., a neural network) can be trained to distinguish “near-tipping” time series from stable time series. EWSNet, developed by Bury et al. [19], is a convolutional neural network trained in this way, which learns to identify combinations of signals in univariate time series and to predict the probability of an impending transition. In tests, EWSNet has detected transitions in complex simulated data and some real data better than traditional individual indicators.
- Models that integrate multiple indicators (“ensemble learning”): Brett & Rohani [77] proposed combining various statistical indicators (e.g., RA, variance) as explanatory variables in a machine learning model (e.g., random forests or logistic regression) to predict a regime change. The premise is that different signals provide complementary information, and a trained algorithm can weigh them optimally. These models address the problem of deciding a priori which indicator to use; instead, they learn from training data which indicators or thresholds are most reliable.

## 4. Applications

In this section, we review notable examples where these methodologies have been applied to real ecosystems, including lakes, coral reefs, grasslands, and marine systems. These successful applications illustrate the opportunities and the challenges of searching EWSs in empirical data.

As an overview of the empirical evidence examined in this section, Table 2 compiles representative case studies in which CSD-based early warning signals have been reported prior to observed critical transitions, listing for each ecosystem the specific signals detected and the key references. The cases span both temporal indicators (rising lag-1 autocorrelation, variance, and skewness) and spatial indicators, and illustrate how the dominant signature varies with ecosystem type; each case is discussed in detail in the corresponding subsection below.

Before turning to ecosystem-specific evidence, it is useful to map each system onto the typology of tipping mechanisms developed in Section 2.6 (B-, N-, R-, and S-tipping). This mapping is not merely taxonomic: as established theoretically in Section 2.5 and Section 2.6, CSD-based indicators are expected to perform well only for B-tipping transitions driven by the gradual approach to a fold (or related) bifurcation and to perform poorly or unreliably for N-, R-, and S-tipping transitions. Pre-classifying the likely tipping mechanism is therefore a prerequisite for both the responsible deployment of EWSs and the interpretation of their successes and failures in the empirical literature reviewed below. Crucially, this classification is not always straightforward from observational data alone: as Boettiger and Batt [110] have shown in a model trophic cascade, the distinction between “state” and “parameter”, and therefore between B- and S-tipping, can depend on ecological details of model formulation that are not always identifiable from time series. Table 3 summarizes the dominant tipping mechanism(s) expected in each ecosystem class, the consequent applicability of classical CSD-based indicators, and the principal confounders to interpretation. Each subsection that follows opens with a brief pointer to its row in Table 3, and the empirical evidence is then assessed in light of these theoretical expectations.

### 4.1. Grasslands, Savannas, and Arid Ecosystems

This ecosystem class falls primarily in the B-tipping regime of Table 3, with secondary R-tipping contributions under rapidly intensifying climate forcing; CSD-based indicators are therefore theoretically expected to apply, with the well-known caveats of non-stationary precipitation and rising forcing variance.

Grasslands, savannas, and arid ecosystems represent paradigmatic systems for studying critical transitions, as they frequently exhibit abrupt shifts between productive vegetated states and degraded configurations under climatic stress or anthropogenic pressure [1,46]. These ecosystems collectively cover approximately 40% of the Earth’s terrestrial surface and support the livelihoods of over two billion people, rendering their stability a matter of profound socioeconomic and ecological importance [111]. Desertification—the transition from semi-arid grassland to bare soil with sparse vegetation—constitutes a particularly consequential form of regime shift, with cascading implications for ecosystem services, food security, carbon sequestration, and regional climate regulation [5,46]. Theoretical vegetation models incorporating soil–water feedbacks, facilitation among plants, and grazing dynamics predict that these systems can undergo fold-type bifurcations, wherein gradual environmental deterioration leads to sudden, catastrophic vegetation collapse once a critical threshold is crossed [46,112]. The existence of alternative stable states implies that recovery following collapse may require substantially more favorable conditions than those that triggered the initial degradation, a phenomenon known as hysteresis [1,113]. These theoretical foundations suggest that early warning signals based on critical slowing down should, in principle, precede catastrophic desertification events, motivating substantial research effort toward their detection and validation.

#### 4.1.1. Theoretical Foundations and Feedback Mechanisms

The theoretical basis for critical transitions in dryland ecosystems derives from scale-dependent feedback mechanisms that can generate and maintain alternative stable states [46,114]. Vegetation patches in water-limited environments modify their local environment in ways that enhance their own persistence: root systems improve soil structure and infiltration capacity, canopy shading reduces evaporative losses, organic matter accumulation increases water retention, and established plants facilitate seedling recruitment through nurse effects [111,114]. These positive feedbacks operate at local scales, creating favorable microsites within vegetation patches.

Simultaneously, negative feedbacks operate at larger scales through resource competition. Vegetation patches deplete soil water from surrounding areas, inhibiting plant establishment in the inter-patch matrix and concentrating resources beneath existing vegetation [46]. The interplay between local facilitation and long-range competition generates the characteristic spatial patterning observed in many dryland ecosystems—banded vegetation (‘tiger bush’), spotted patterns, and labyrinthine configurations—that reflect self-organized responses to water limitation [115,116].

Mathematical models incorporating these feedbacks predict that as environmental stress increases (reduced precipitation, elevated grazing pressure, or soil degradation), the system approaches a fold bifurcation. Near this threshold, the dominant eigenvalue governing vegetation dynamics approaches zero, generating critical slowing down that should manifest as rising autocorrelation and variance in vegetation cover time series [37,38]. Additionally, the spatial structure of vegetation is predicted to undergo systematic changes—increased spatial variance, altered patch-size distributions, and modified connectivity patterns—that may serve as complementary spatial early warning signals [35].

#### 4.1.2. Spatial Early Warning Signals

A distinctive feature of EWS research in dryland ecosystems is the prominence of spatial indicators, reflecting the strong spatial organization imposed by scale-dependent feedbacks [35,36]. Kéfi et al. [5] provided seminal evidence that spatial vegetation patterns can serve as indicators of the ecosystem degradation state. Analyzing vegetation cover data across a degradation gradient in Mediterranean arid ecosystems, they demonstrated that patch-size distributions deviated systematically from power-law scaling in degraded sites, suggesting that spatial pattern analysis could diagnose proximity to critical thresholds.

Subsequent theoretical work formalized the connection between critical slowing down and spatial pattern changes. As systems approach fold bifurcations, critical slowing down manifests not only in temporal dynamics but also in spatial structure: spatial variance increases as vegetation becomes patchier, spatial autocorrelation rises as patches become more clustered, and characteristic length scales shift as the dominant spatial modes change [36]. Kéfi et al. [35] provided a comprehensive methodological framework for computing spatial EWSs, demonstrating their application to both simulated and empirical vegetation data.

The theoretical prediction that spatial patterns undergo systematic changes before desertification has received support from multiple empirical studies. In the Sahel region of Africa, analyses of vegetation pattern dynamics have revealed that degrading sites exhibit reduced patch connectivity, altered patch-size distributions, and increased spatial variance compared to stable sites [5,117,118]. Importantly, these spatial changes may be detectable even when long temporal records are unavailable, offering practical advantages for monitoring programs in data-limited regions [5,117].

However, the relationship between spatial pattern and degradation state is not always straightforward. Multiple spatial pattern types (gaps, spots, and bands) can be stable under identical environmental conditions, complicating the use of pattern type alone as a degradation indicator [119,120]. Furthermore, some spatial pattern changes may reflect adaptive reorganization that enhances resilience rather than approaching collapse, and distinguishing these cases remains an active area of research [120,121].

#### 4.1.3. Remote Sensing Evidence and Temporal Indicators

The advent of long-term satellite observation has enabled investigation of early warning signals across extensive arid and semi-arid regions, providing unprecedented opportunities to test theoretical predictions at landscape to continental scales [122]. Vegetation indices derived from satellite imagery—particularly the Normalized Difference Vegetation Index (NDVI) and related spectral measures—provide consistent, spatially comprehensive time series of vegetation productivity that can be analyzed for EWSs.

Verbesselt et al. [103] conducted a landmark analysis of remotely sensed resilience across tropical and subtropical vegetation, including extensive savanna and dryland regions. Examining satellite vegetation index time series spanning 1982–2012, they quantified resilience through recovery rates following perturbations (primarily drought events). Their analysis revealed that in areas vulnerable to transition, the rate of vegetation recovery following rainfall deficits diminished over time—a direct manifestation of critical slowing down detectable in remotely sensed data. Crucially, areas showing declining recovery rates were more likely to subsequently experience permanent vegetation loss, providing prospective evidence that satellite-derived resilience indicators can anticipate degradation.

More recent analyses have extended these findings to African dryland ecosystems. Barlow et al. [123] quantitatively monitored the resilience of patterned vegetation in the Sahel using Sentinel-2 imagery, demonstrating that morphological analysis of vegetation pattern classes can distinguish resilience levels across sites and that AR(1) and variance are sensitive to pattern morphology and precipitation gradients. Veldhuis et al. [102] demonstrated spatial slowing down in satellite vegetation patterns before desertification transitions in East African rangelands, with year-to-year changes in vegetation cover decreasing in areas approaching degradation thresholds—consistent with critical slowing down predictions. Similarly, Forzieri et al. [122] documented declining forest resilience globally using satellite observations, including pronounced resilience loss in semi-arid woodland systems approaching apparent tipping points.

Temporal EWSs in dryland time series have also been investigated using traditional statistical indicators. Analyses of simulated vegetation dynamics under increasing stress have consistently shown rising autocorrelation and variance in vegetation cover preceding collapse [35,38]. At the landscape scale, remote sensing has facilitated the investigation of early warning signs in arid regions. Verbesselt et al. [103] examined satellite vegetation index time series in tropical forests and savannas, revealing that in areas vulnerable to transition (from savanna to desert or from forest to savanna), the rate of vegetation recovery following rainfall events diminished after years of recurrent drought. Similarly, more recent studies conducted in dry African ecosystems have identified spatial slowing, characterized by decreased year-to-year changes in cover, as well as reddening in the inter-annual fluctuations of primary productivity prior to sudden degradation events. Notably, Veldhuis et al. [124] provided evidence of spatial slowing down in satellite vegetation patterns before desertification transitions, thereby supporting theoretical predictions.

In temperate grasslands characterized by seasonal climates, it is challenging to discern ecological signals because of pronounced seasonal and successional variability. However, Clements and Ozgul [125] demonstrated that even biological traits, such as the average body size of herbivores, can shift prior to population collapse within grassland ecosystems. Specifically, a decrease in size variance, indicating a more homogeneous group of individuals, preceded significant reductions in some populations. This phenomenon is interpreted as a loss of demographic resilience.

In grasslands and arid ecosystems, EWSs are supported by modeling and remote sensing results; however, direct field time-series evidence is less prevalent than in lakes. Spatial indicators often provide more pronounced insights than temporal indicators, as exemplified by the formation of patchy vegetation patterns, which are considered an early warning sign of potential desertification. The integration of satellite data with on-site monitoring remains a promising approach for improving the early detection of degradation in these ecosystems. However, empirical validation using real vegetation time series has yielded mixed results. Génin et al. [126] developed the spatialwarnings R package to facilitate systematic computation of spatial EWSs in vegetation data; while spatial indicators showed expected patterns in some datasets, others exhibited weak or inconsistent signals, highlighting the influence of data quality, environmental heterogeneity, and non-stationarity on EWS detection.

The heterogeneity of results across remote sensing studies reflects a broader challenge common to all temporal EWS analyses in drylands: non-stationarity, environmental gradients, and confounding land-use change can generate trends in AR(1) and variance unrelated to genuine loss of ecological resilience. These results reinforce the need to combine temporal indicators with spatial diagnostics and careful detrending of trends and seasonality [35,38].

#### 4.1.4. Savanna–Forest Transitions

Beyond desertification, dryland ecosystems encompass savanna–forest boundaries that can exhibit critical transitions in both directions [48]. The distribution of tropical vegetation between closed-canopy forest and open savanna states appears bimodal at intermediate precipitation levels, suggesting alternative stable states maintained by fire–vegetation feedbacks [48]. Forests suppress fire through shading (reducing grass fuel loads) and maintaining high canopy moisture, while savannas promote fire through continuous grass cover and seasonal drying. This feedback can maintain sharp boundaries between adjacent forest and savanna, with transitions between states occurring abruptly when thresholds are crossed.

Early warning signals for savanna–forest transitions have been investigated primarily through fire regime dynamics and vegetation structure indicators. Increasing fire frequency beyond historical ranges can progressively erode forest resilience by depleting seed banks, eliminating fire-sensitive species, and reducing canopy cover, potentially pushing the system toward savanna dominance. Time-series analyses of burned area dynamics in transitional forest–savanna ecotones have revealed that elevated interannual variability in fire extent often precedes vegetation state changes, consistent with theoretical expectations of increased variance near bifurcations [37].

Furthermore, shifts in the coupling between fire behavior and environmental drivers have been identified as potential indicators of declining forest resilience. In stable forest systems, internal feedback mechanisms—including high canopy moisture content, reduced understory fuel loads, and humid microclimatic conditions—buffer fire regimes against climatic fluctuations, dampening correlations between burned area and drought severity. As forests lose resilience and approach transition thresholds, this buffering capacity diminishes, resulting in increasingly tight coupling between fire extent and exogenous drivers. Empirical observations from Amazonian and African forest–savanna boundaries have documented this phenomenon, with increasing fire–climate correlations serving as harbingers of subsequent savanna encroachment.

#### 4.1.5. Trait-Based and Demographic Indicators

Beyond aggregate vegetation indices, biological traits and demographic structure may shift prior to population collapse within grassland and savanna ecosystems, offering complementary indicators of declining resilience. Clements and Ozgul [125] demonstrated that trait-based indicators—including body size distributions of herbivore populations—can provide early warning of impending collapse. In their analysis, a decrease in body size variance (indicating demographic homogenization) preceded population collapse in grassland herbivores. This pattern reflects loss of demographic resilience: populations with diverse age and size structure can buffer environmental variability through differential responses among individuals, while homogenized populations lack this buffering capacity.

Similar principles may apply to plant communities, where diversity in functional traits (drought tolerance, rooting depth, and phenology) confers resilience to environmental fluctuations. Declining functional diversity in grassland plant communities under chronic stress could potentially serve as an early warning signal, though this hypothesis remains less thoroughly tested than trait-based indicators in animal populations.

Demographic early warning signals have also been proposed for long-lived species in savanna ecosystems. Changes in tree population age structure—particularly reduced recruitment and loss of juvenile cohorts—may indicate declining population viability before changes in adult abundance become apparent. For slow-growing woody species, demographic indicators may provide earlier warning than aggregate biomass or cover measures, as changes in recruitment can precede detectable changes in standing biomass by years to decades.

#### 4.1.6. Case Studies and Empirical Evidence

Several well-documented case studies illustrate both the promise and challenges of EWS detection in dryland ecosystems.

Sahel Greening and Degradation. The Sahel region has experienced dramatic vegetation changes over recent decades, including both degradation (1970s–1980s droughts) and partial recovery (‘re-greening’ since the 1990s). Analyses of satellite vegetation time series have revealed spatial heterogeneity in recovery patterns, with some areas showing sustained improvement while others remain degraded or continue declining [127,128]. Preliminary analyses suggest that pre-drought spatial vegetation patterns differed between resilient and degraded sites, though confounding factors (soil type, land use history, and topography) complicate interpretation.
Australian Rangelands. Long-term monitoring data from Australian arid rangelands have revealed episodic vegetation state changes associated with drought and grazing pressure. Analyses of these transitions have documented hysteresis [129], conditions required for recovery exceed those that triggered collapse, supporting alternative stable state dynamics. Systematic analysis of whether EWSs preceded these documented transitions remains limited by data availability and the challenge of distinguishing gradual degradation from abrupt regime shifts.
Mediterranean Shrublands. Mediterranean ecosystems have provided important case studies for spatial EWS research. Degradation gradients across environmental or land-use intensity gradients offer space-for-time substitution approaches, wherein sites at different degradation stages are compared to infer temporal dynamics [5]. These analyses have revealed systematic changes in spatial vegetation patterns along degradation gradients, supporting the potential utility of spatial EWSs [35], though true prospective tests of predictive capacity remain rare.

Taken together, these case studies illustrate both the promise and difficulty of operationalizing EWSs in dryland systems. The Sahel, Australian rangelands, and Mediterranean drylands all provide evidence for hysteresis and spatial-pattern shifts across degradation gradients; however, truly prospective, out-of-sample evaluations of predictive EWS performance remain comparatively rare [102]. This gap between retrospective detection and genuine prospective prediction is one of the most pressing priorities for future research in this ecosystem type. Combining temporal indicators (AR(1), variance, and recovery rates) with spatial diagnostics, and carefully treating non-stationarity and confounding environmental drivers, represents the most robust analytical pathway currently available [35,38].

### 4.2. Lakes and Freshwater Aquatic Systems

Shallow lakes are the canonical B-tipping system in the EWS literature (Table 3), and, accordingly, they are the strongest theoretical expectation of CSD applicability among the ecosystems reviewed here.

Shallow lakes are classic systems for studying critical transitions. The prototypical example is the change of a lake from clear water (dominated by macrophytes) to turbid water (dominated by phytoplankton) due to nutrient eutrophication. Scheffer et al. theorized that this system undergoes a fold bifurcation with hysteresis, and subsequent studies sought early warning systems (EWSs) before the loss of clarity [44]. Wang et al. analyzed paleolimnological (sediment) data from a lake [81]. They found evidence of flickering years before the definitive transition to a turbid state: that is, fluctuations oscillating between clear and turbid conditions, reflected in greater variance and skewness in nutrient indicators. This flickering served as a robust early warning of the imminent collapse. On the other hand, controlled ecosystem-scale experiments have also been enlightening: Carpenter et al. manipulated a Wisconsin lake by gradually increasing piscivory pressure, leading to a regime shift. Using water quality and planktonic population data, they observed increasing variance and autocorrelation as the critical point was approached, which was consistent with the expected critical slowdown [101]. These findings highlight the utility of statistical indicators, such as variance and autocorrelation, in detecting critical slowing down prior to regime shifts. Moreover, they emphasized the importance of integrating empirical data and experimental manipulation to understand the resilience of ecosystems. These approaches provide valuable insights for developing effective management strategies to prevent undesirable ecosystem changes.

However, efforts to detect EWSs in natural lakes have not always been consistently successful. A recent comprehensive study analyzed time-series data of phytoplankton and zooplankton from nine lakes worldwide, using various metrics (both univariate and multivariate) to identify early warnings preceding documented historical changes. The findings revealed that traditional signals (AR(1); variance) were only effective in predicting some change events, while in other instances, they either triggered false alarms or failed to detect any changes at all [19]. The study concluded that EWSs have limited applicability to empirical lake data owing to complicating factors such as external noise, seasonal influences, and low temporal resolution, which obscure clear trend detection. In certain lakes, the observed transitions were non-critical (e.g., abrupt changes caused by point disturbances rather than bifurcations) and, as anticipated, did not show prior critical slowing [130]. Despite these challenges, the study also noted that combining methods (such as multivariate indicators and machine learning tools like EWSNet [131]) modestly enhanced predictive capacity, although no single method achieved high predictive certainty on its own.

In lotic ecosystems, such as rivers and streams, clear examples of EWSs are less common because of the open nature of these environments. However, certain controlled trophic cascades in experimental streams have been reported to produce EWSs. For instance, variations in dissolved oxygen levels and turbidity have been investigated as potential early indicators of hypoxia in eutrophic estuaries. Shallow lakes have effectively functioned as "natural laboratories" for validating EWSs, demonstrating notable success in predicting water quality declines. Nevertheless, these studies underscore the practical challenges associated with the use of real-world ecosystem data. Factors such as the length of data records (many lakes have limited historical data) and external climatic influences, such as harsh winters and droughts, can obscure general trends.

### 4.3. Coral Reefs

Unlike lakes, coral reefs fall primarily in the S-tipping regime (Table 3): most documented transitions are driven by acute bleaching events, storms, or disease outbreaks rather than by gradual parameter drift. Classical CSD indicators are therefore expected to perform weakly, and the empirical evidence reviewed below is consistent with this expectation [110].

Coral reefs can experience sudden shifts from being dominated by live coral to being overrun by macroalgae or other unwanted organisms such as debris or cyanobacteria. These regime changes are often triggered by disturbances such as bleaching events caused by thermal stress, overfishing of herbivores, or pollution, and pose a significant concern for tropical marine ecology [132,133,134].

Theoretical models suggest that coral reefs exhibit non-linear dynamics, including feedback loops. For example, healthy corals create conditions that favor their growth, such as high structural complexity and successful recruitment, whereas algal dominance hinders coral recovery. This dynamic can lead to alternative stable states—either a thriving coral reef or a degraded algae-dominated reef.

#### 4.3.1. Empirical Evidence and Detection Challenges

However, one major challenge is the scarcity of long-term reef monitoring, which can be influenced by confounding factors such as hurricanes and ocean variability. Despite these challenges, some local time-series studies have reported subtle signs of such instability. For instance, prior to a decline in coral cover, an increase in the interannual variability of short algae (less than 5 cm) within the community was observed in the present study. This increase was interpreted as a sign of increasing instability. Similarly, some studies have noted increased temporal autocorrelation in coral cover measurements taken every few months just before mass mortality events, although these findings were not statistically significant [135].

Dakos et al. [63] proposed that generic indicators of CSD could be used as a basis to rank coral reefs by resilience, in the sense that reefs approaching a tipping point should already exhibit statistically detectable trends in variance and/or autocorrelation in relevant time series. In this framing, reefs “closer to the threshold” are expected to show increasingly persistent deviations from their baseline state, reflecting a weakening ability to recover from perturbations, whereas more resilient reefs should display comparatively stable fluctuation statistics. However, despite the conceptual appeal of this approach, dedicated empirical tests remain relatively scarce, and operationalizing CSD for reefs has proven difficult [136].

In the Great Barrier Reef (Australia), retrospective analyses of coral cover percentage data suggest that following repeated bleaching events, coral recovery slowed (a slowdown in cover dynamics), and spatial variance between sites increased before an algae-dominated regime was established in certain reef sections. This aligns with the expectation that spatially variable resilience can provide a signal (some patches collapse sooner than others, increasing variance between sites) [136,137].

#### 4.3.2. Disturbance-Mediated Transitions and Hybrid EWS Approaches

While evidence from reefs continues to develop, there is cautious optimism that combining multiple indicators, such as the integrity of herbivorous fish food webs and fluctuations between coral and algae, could help predict a critical tipping point. For instance, a reduction in herbivore functional diversity, including the loss of keystone species, may serve as an early “biomarker” signaling that the ecosystem is at risk of losing control over algae growth. Integrative ecological web approaches, along with early warning systems (EWSs), are currently being developed [134,138].

A central obstacle is that many reefs do not experience a clean, gradual drift toward collapse driven by a slowly changing control parameter—the canonical setting where CSD theory is most straightforward. Instead, reefs often respond to acute, pulse-like disturbances such as abrupt bleaching triggered by short-lived marine heatwaves, storm damage, or disease outbreaks. From a time-series perspective, these events behave less like the smooth “ramping” of a parameter and more like impulsive shocks superimposed on a noisy background. Such shocks can dominate the observed dynamics, masking or even breaking the assumptions under which classic CSD indicators are expected to rise monotonically. In other words, a reef may flip states because it is hit hard, not necessarily because it has been drifting slowly toward instability in a way that produces textbook early warning patterns.

However, the absence of a slow parameter drift does not imply the absence of informative precursors. Even under disturbance-driven dynamics, reefs can exhibit signatures of reduced stability that are consistent with the system moving closer to the boundary between alternative states. One such signature is flickering, in which the system intermittently visits an alternative regime before settling permanently. In the Caribbean, for instance, some sites have exhibited episodes of transient algal dominance in the years preceding a long-lasting shift to algae following major coral mortality. These brief algal blooms subsequently receded as corals partially recovered, indicating that the coral-dominated state was becoming increasingly fragile, and small perturbations were sufficient to push the system into an algal-like configuration, even if it could still “snap back” for a time. Interpreted through the lens of dynamical systems, flickering suggests that the reef’s trajectory was increasingly influenced by the competing basin of attraction, with the system hovering near a threshold where either coral or algae could temporarily prevail over the other [72,81].

These considerations suggest a useful refinement to the original CSD-based classification idea: reef resilience assessment may benefit from combining classical CSD metrics (variance; autocorrelation) with transition-focused precursors, such as flickering and other indicators of bistability. This hybrid perspective acknowledges that reef degradation is often disturbance-mediated while still leveraging the broader principle that declining resilience leaves statistical fingerprints in ecological time series, even if those fingerprints are not always expressed as smooth, monotonic increases in variance and AR(1).

### 4.4. Marine Fisheries and Pelagic Ecosystems

Marine fisheries represent one of the most consequential domains for early warning research: stock collapses carry severe ecological and socioeconomic consequences yet have historically been detected only after irreversible demographic damage has occurred [139]. Pelagic ecosystems—the open-ocean communities of plankton, forage fish, and their predators—present a related but distinct challenge, in which abrupt community reorganizations are often driven by multi-decennial oceanographic forcing rather than by the gradual parameter drift that generates classical critical slowing down. Together, these systems test the boundaries of the EWS framework more severely than any other ecosystem type reviewed here, because the tipping mechanisms involved span the full typology of Section 2.6: fold bifurcations in overexploited stocks, rate-induced and shock-induced transitions in plankton regimes, and multispecies reorganizations that combine elements of both.

#### 4.4.1. Theoretical Basis for Tipping in Marine Systems

Fish stock dynamics can exhibit fold bifurcations when density-dependent recruitment processes interact with harvesting mortality. The canonical mechanism involves a dispensatory (Allee-effect) recruitment function: at low stock sizes, per-capita recruitment declines because mate-encounter rates fall, schooling anti-predator defenses erode, or cooperative spawning behavior is disrupted [1,106]. When a slowly increasing harvest rate erodes stock size toward the Allee threshold, the dominant eigenvalue of the linearized stock-recruitment system approaches zero from below, generating CSD in exactly the form described in Section 2.3. The associated EWS predictions are unambiguous: rising variance and autocorrelation in biomass or recruitment indices, increasing skewness toward low-biomass states, and slowing recovery rates following perturbations should all precede collapse.

A second, distinct mechanism operates through regime shifts in the broader marine ecosystem. In upwelling systems, semi-enclosed seas, and continental shelf ecosystems, multi-decennial oceanographic oscillations can abruptly reorganize productivity regimes, shifting the carrying capacity and recruitment environment for entire fish communities simultaneously [107,140]. These transitions often resemble rate-induced or shock-induced tipping—driven by rapid atmospheric forcing or sudden upwelling changes—rather than the gradual bifurcation approach that generates CSD. These indicators provide no reliable warning of R- or S-tipping events; this distinction has immediate practical implications for EWS design in fisheries and pelagic systems.

#### 4.4.2. Empirical Evidence and Case Studies

Atlantic cod and North Atlantic groundfish. (Retrospective.) The collapse of Northwest Atlantic cod stocks in the early 1990s has been subjected to multiple retrospective EWS analyses. Examination of stock assessment time series spanning 1960–1992 documented rising interannual variance in recruitment indices and increasing lag-1 autocorrelation in spawning stock biomass in the decade preceding collapse, consistent with CSD predictions [15,141]. However, Boettiger and Hastings [23] demonstrated that these apparent warning trends are statistically indistinguishable from noise expected under a null hypothesis of no approaching bifurcation when appropriate surrogate tests are applied. Because the analysis was conducted retrospectively on a series selected precisely because it ended in a known collapse, conditional sampling bias cannot be excluded as a partial explanation for the apparent signals [23,142]. The cod case therefore illustrates both the theoretical plausibility of fisheries’ EWSs and the inferential limitations that constrain their operational value when tested retrospectively without pre-specified null models.
Baltic Sea food-web reorganization. (Retrospective; quasi-prospective.) The Baltic Sea has provided one of the most thoroughly analyzed multispecies case studies for EWSs in marine systems. Möllmann et al. [140] documented a major reorganization of the Baltic food web in the late 1980s, involving simultaneous regime shifts in cod, sprat, herring, and zooplankton communities driven by the interaction of fishing pressure and eutrophication. Subsequently, Lindegren et al. [143] applied CSD-based EWSs to multivariate Baltic monitoring data and demonstrated that rising variance and autocorrelation in aggregate community indicators—particularly zooplankton biomass and fish recruitment composites—anticipated the reorganization by three to five years. Crucially, this analysis was structured as a quasi-prospective evaluation: indicators were computed on data available up to a pre-specified decision point, with the transition used as the validation event rather than the analysis target. This design provides stronger evidence for predictive validity than a purely retrospective analysis, though it falls short of a true prospective test because the transition outcome was known when the study was designed. The Baltic case is presently the strongest available evidence for operational EWS performance in a marine multispecies context.
North Pacific productivity regime shifts. (Retrospective.) Litzow and Mueter [107] assessed ecological regime shifts in the North Pacific, showing that abrupt, synchronized shifts in fish community structure were preceded by increasing temporal variance and spectral reddening in productivity indices. Hsieh et al. [108] showed that fishing pressure itself elevates interannual variability in exploited stocks, independently of proximity to a bifurcation. This finding creates a systematic false-positive risk specific to fisheries: the primary management action (fishing) directly inflates the primary EWS indicator (variance), a confounding effect with no direct analogue in lake, forest, or grassland applications. All analyses in this case were retrospective, relying on archived stock assessment records; no prospective or experimental evaluation has been conducted.
Global stock assessment survey. (Retrospective.) Vert-Pre et al. [144] conducted the most comprehensive retrospective EWS analysis available, examining 230 fish stock time series from the RAM Legacy database for evidence of productivity regime shifts. Variance-based indicators correctly anticipated approximately half of documented collapses, but false-positive rates were substantial (approximately 40% of flagged stocks did not subsequently collapse). Anderson et al. [145] reached similar conclusions: EWSs in fisheries have meaningful but limited predictive power, particularly for stocks subject to externally driven regime shifts rather than endogenous bifurcation dynamics. The retrospective design of both studies, and the fact that the stock assessment records used were themselves model-derived rather than directly observed, introduce inferential layers that limit conclusions about genuine predictive validity.

Peruvian anchovy and the Humboldt Current system. (Retrospective.) The collapse of the Peruvian anchoveta (*Engraulis ringens*) stock in the early 1970s, associated with the 1972–1973 El Niño event, represents one of the most dramatic fishery collapses on record and provides an instructive contrast to the cod case. Unlike the Northwest Atlantic groundfish system, where chronic overharvesting gradually eroded stock biomass toward an Allee threshold consistent with B-tipping, the anchoveta collapse was primarily driven by the rapid shoaling of the thermocline and catastrophic reduction in upwelling productivity triggered by the El Niño anomaly, a dynamics consistent with S- or R-tipping rather than a gradual bifurcation approach [139]. Retrospective analyses of recruitment indices and sea surface temperature anomalies have not identified systematic pre-collapse trends in variance or autocorrelation that would constitute classical CSD signatures, consistent with theoretical expectations for externally forced transitions. The anchoveta case therefore illustrates the practical consequence of the B/R/S-tipping distinction (Section 2.5): deploying CSD-based EWSs in upwelling systems subject to strong ENSO forcing carries a high risk of both false negatives (genuine collapses missed because no CSD signal precedes them) and false positives (elevated variance during La Niña recovery phases misinterpreted as resilience loss). This system-specific confounding underscores the need to incorporate oceanographic forcing indices as covariates when interpreting EWS indicators in eastern boundary current fisheries.

Pelagic plankton regime shifts. (Retrospective.) Hsieh et al. [108] documented that spectral reddening of climate–biological variability and rising variance in plankton indices preceded regime shifts in several pelagic systems, while Conversi et al. [109] provided a holistic synthesis demonstrating that synchronization of previously anti-phase population oscillations anticipated species-level collapses in open-ocean communities. Both analyses relied on long-term plankton monitoring archives (Continuous Plankton Recorder and equivalent programs), making them among the longest available marine time series. A distinctive feature of approaching pelagic regime shifts may therefore be increasing synchronization among previously independent population fluctuations: as the dominant eigenvalue approaches zero near a bifurcation, all system components become increasingly responsive to the same slow mode of variability, consistent with the multivariate CSD predictions developed in Section 5.2.1 However, the dominant tipping mechanism in pelagic systems involves rapid atmospheric forcing and inter-basin oceanographic teleconnections that are more consistent with R-tipping than B-tipping, and, variance and autocorrelation are expected to perform poorly under R-tipping dynamics. The empirical detection of spectral reddening in these systems may therefore reflect changing external forcing rather than genuine CSD, a distinction that retrospective analyses cannot easily resolve.

#### 4.4.3. Implications for Predictive Validity

Three lessons emerge from the marine and pelagic evidence that apply broadly to the EWS field. First, the distinction between endogenous B-tipping and exogenous R- or S-tipping is not merely theoretical: it determines whether CSD-based indicators have any predictive validity at all, and pre-classifying the likely tipping mechanism before deploying EWSs is a prerequisite for responsible application [145]. Second, fishing pressure confounds variance-based indicators in a system-specific way that demands explicit null-model construction; any fishery’s EWS analysis should compare observed trends against surrogates generated under a null hypothesis of a stable but heavily fished stock [39]. Third, no marine EWS study has yet achieved a true prospective test under operational conditions—one in which thresholds were pre-specified, decisions were made, and predictive accuracy was evaluated against subsequent outcomes. This gap is the central unresolved challenge in marine EWSs, as it is across all ecosystem types reviewed here.

The pelagic case raises an additional point of operational significance: long-term plankton surveys such as the Continuous Plankton Recorder in the North Atlantic provide the only extended time series suitable for EWS analysis at basin scales, and the integration of EWS indicators into ocean observing systems could in principle provide advance warning of impending regime shifts with consequences for fisheries, carbon cycling, and marine ecosystem services. However, the large spatial scales, complex forcing mechanisms, and limited mechanistic understanding of pelagic transitions mean that EWSs in these systems should be interpreted cautiously. The challenge of prospective prediction, demonstrating that EWSs provide useful advance warning rather than merely retrospective confirmation, remains largely unmet for basin-scale ocean regime shifts, and this gap is one of the central unresolved issues in marine EWS research.

### 4.5. Forests

Forest ecosystems span multiple tipping mechanisms (Table 3): B-tipping at the landscape scale via vegetation–climate feedbacks (e.g., Amazonian dieback), R-tipping under rapid drying, and S-tipping through fire and drought pulses. CSD-based indicators are most reliably detected at the landscape scale via remote sensing, and degrade at the stand scale where demographic lags dominate.

Forests can exhibit critical transitions on large scales, such as the savannization of the Amazon (collapse from humid forest to dry savanna) under climate change or the mass mortality of boreal forests, converting them into steppes after droughts and pest outbreaks. Such changes often involve hysteresis (e.g., once a forest is lost, local aridity increases, preventing regeneration). Identifying ecosystem services (ESSs) in forests is complicated by the longevity of trees and the slowness of dynamics, but there are notable studies. Verbesselt et al. [103] used satellite Normalized Difference Vegetation Index (NDVI) time-series (1988–2012) to detect signs of resilience loss in tropical forests. They found that in areas of the Amazon that subsequently experienced permanent biomass decline, the NDVI showed increasing autocorrelation and variance approximately 2–3 years before the transition [103]. Following severe drought events, areas that did not recover showed “scars” in the series: greater persistence at low values (high AR) and anomalous fluctuations. This is one of the first examples of an EWS applied to forests on a continental scale.

The detection of EWSs in forest ecosystems increasingly relies on time-series analyses of demographic structures, particularly age and size distributions, to anticipate critical transitions and regime shifts. Theoretical frameworks predict that as forest populations approach tipping points, characteristic changes in demographic structure will emerge, including the simplification of age-class distributions and the loss of recruitment cohorts [37,38]. For instance, the persistent absence of seedlings and saplings following disturbance events may indicate regeneration failure, signaling that the system is approaching a critical threshold beyond which recovery to the original state is unlikely [146]. Empirical applications of these principles have been documented in diverse forest biomes. In boreal forests, prolonged recruitment gaps following fire disturbances have been interpreted as potential indicators of imminent state changes, where forests may transition to alternative stable states, such as shrublands or grasslands, rather than regenerating to their pre-disturbance condition [147]. Similarly, in Mediterranean ecosystems, time-series analyses of stand structures have revealed declining regeneration rates that precede drought-induced forest dieback events [148].

Beyond demographic indicators, complementary EWS metrics derived from time-series data have strengthened the predictive capacity for detecting approaching transitions in forest systems. Critical slowing down, the phenomenon whereby systems recover more slowly from perturbations as they approach bifurcation points, has been detected through increasing temporal autocorrelation and variance in tree growth chronologies and forest productivity indices [16,35]. Studies in temperate forests have demonstrated that rising autocorrelation in radial growth time-series can precede widespread mortality events by several years to decades, providing a potential operational window for management interventions [149]. Furthermore, analyses of remotely sensed vegetation indices across tropical and temperate forests have revealed spatial and temporal signatures consistent with approaching tipping points, including increased flickering between states and the propagation of recovery delays across landscapes [103,122]. Collectively, these findings underscore the utility of integrating demographic monitoring with time-series-based EWS approaches to enhance our capacity for forecasting critical transitions in forest ecosystems under accelerating global change pressures.

Fire-regime dynamics also constitute an important class of EWSs for the forest–savanna boundary; because the relevant feedbacks (fire–vegetation coupling, interannual variance in burned area, and tightening of fire–climate correlations on the approach to transition) are mechanistically tied to the dryland vegetation feedbacks treated earlier, this material is developed in Section 4.1.4 and is not repeated here.

#### Remote Sensing Applications and Climate-Vegetation Feedbacks: EWS for Large-Scale Forest Collapse

Global vegetation models incorporating climate–forest feedbacks have provided compelling theoretical evidence that large-scale forest systems, particularly the Amazon basin, may exhibit detectable EWSs years to decades before catastrophic collapse. These models integrate critical biophysical processes, including evapotranspiration regulation, precipitation recycling, and moisture-dependent fire dynamics, which generate positive feedback loops capable of driving abrupt transitions between forested and degraded states [2,150,151]. Model projections suggest that as deforestation and climate change progressively weaken these self-reinforcing mechanisms, characteristic EWSs will emerge across spatial and temporal domains. Specifically, increasing spatial autocorrelation in seasonal drought patterns has been predicted as a precursor to basin-wide dieback, reflecting the loss of moisture recycling capacity and homogenization of water stress across previously heterogeneous landscapes [151,152]. Additionally, “flickering” dynamics, whereby satellite imagery reveals transient mosaics of intact forest patches alternating with degraded clearings, have been identified as potential signatures of bistability, indicating that the system oscillates between alternative attractors as it approaches a tipping point [37,38]. Recent analyses of long-term satellite records have detected such patterns in portions of the southeastern Amazon, where increasing dry-season severity and fire frequency have driven localized state changes consistent with the model predictions [104,153].

The operationalization of EWS detection through remote sensing platforms represents a particularly promising frontier for anticipating forest regime shifts at regional to continental scales. Despite the inherent inertia of forest ecosystems, which are characterized by long tree lifespans, slow demographic turnover, and lagged responses to environmental forcing, satellite-derived vegetation indices have demonstrated the capacity to capture subtle signatures of declining resilience before overt structural degradation becomes apparent [103,122]. Critical slowing down, manifested as the delayed recovery of vegetation greenness (e.g., NDVI; EVI) or above-ground biomass proxies following drought episodes or disturbance events, has emerged as one of the most robust and practically measurable indicators of approaching tipping points [16]. Time-series analyses of recovery rates across tropical, temperate, and boreal forests have revealed that declining resilience, quantified as lengthening return times to baseline conditions, often precedes widespread mortality and canopy loss by several years, providing a potential early detection window for management and policy intervention [103,122]. These advances underscore the transformative potential of integrating mechanistic vegetation models with satellite-based EWS monitoring frameworks to anticipate ecological tipping points under accelerating climate change, offering critical lead times for adaptation strategies aimed at averting regional-scale forest collapse and its associated impacts on the carbon cycle, biodiversity, and socioeconomic consequences.

Because the reliability of critical-slowing-down (CSD) indicators depends on *how* a system tips, it is useful to organize candidate ecosystems by their dominant transition mechanism before assessing where temporal early warning signals (EWS) are likely to succeed or fail. Table 3 summarizes five representative ecosystem classes, linking each to the mechanism(s) expected to govern its transitions (bifurcation-, noise-, rate-, or shock-induced.

## 5. Perspectives on the Use of EWS in Ecology

The capacity to anticipate critical transitions in ecological systems before they occur represents one of the most consequential challenges in contemporary ecology. This section examines how early warning signals (EWSs) derived primarily from time-series analysis are transforming our approach to ecosystem management, the methodological advances that are expanding the EWS toolkit, and the research frontiers that will shape the next generation of resilience diagnostics.

### 5.1. From Reactive Management to Anticipatory Risk Assessment

Conventional ecological monitoring has long operated in a reactive mode, detecting ecosystem deterioration only after critical thresholds have been crossed—a point at which recovery is often slow, expensive, or altogether infeasible. The EWS paradigm fundamentally reorients this approach by seeking to identify loss of resilience prior to regime shifts through the detection of statistical signatures of destabilization in time-series data [37,63]. The theoretical foundation for this approach rests on the phenomenon of critical slowing down (CSD): as a system approaches a bifurcation point, its rate of recovery from small perturbations decreases, leaving characteristic imprints in temporal dynamics that can be quantified through indicators such as rising lag-1 autocorrelation and increasing variance [74].

The translation of this theoretical insight into operational practice is most naturally achieved through resilience dashboards embedded within routine monitoring programs. In such frameworks, CSD-based indicators are computed in rolling windows over time series and reported alongside uncertainty estimates derived from null model comparisons [63]. Crucially, these dashboards should not be conceived as binary alarm systems but rather as decision-support layers that integrate multiple lines of evidence: indicator trends extracted from temporal data, mechanistic understanding of pressures and feedbacks, and context-specific assessments of vulnerability. This parallels the evolution of meteorological forecasting, which translates complex model outputs into probabilistic risk communications rather than deterministic yes/no predictions.

For time-series-based EWSs to inform governance effectively, however, careful attention must be paid to operational thresholds. Policy and management decisions typically require actionable categories (e.g., traffic-light risk levels), whereas temporal EWS outputs are inherently continuous and subject to noise. A productive direction therefore involves defining thresholds explicitly tied to management objectives—such as acceptable false alarm probabilities or expected loss functions—rather than relying solely on generic statistical significance. This naturally leads to the probabilistic and Bayesian decision frameworks discussed in subsequent sections, where uncertainty is treated as a first-class output and interventions are evaluated under explicit risk models [63].

### 5.2. Methodological Advances in Time-Series-Based EWS

The early EWS toolkit, centered on variance and autocorrelation computed from univariate time series, has undergone substantial expansion. This methodological evolution reflects two key recognitions: that no single indicator is universally reliable, and that many real-world transitions deviate from the classical CSD-at-a-bifurcation narrative. The advances described below constitute what may be termed a second generation of EWS methods—approaches that are multi-source, probabilistic, and explicitly designed to accommodate nonstationarity and observational limitations.

#### 5.2.1. Multivariate and Network-Based Methods

Ecosystems are inherently multicomponent systems characterized by correlated species dynamics, functional redundancy, and cross-scale coupling. Resilience loss may therefore be distributed across communities and trophic levels in ways that are not strongly expressed in any single state variable. Multivariate EWS approaches synthesize information across multiple time series, exploiting the observation that destabilization may manifest as changes in covariance structure, shifts in dominant eigenvalues of community matrices, or alterations in coherence patterns that are more sensitive than univariate summaries in high-dimensional settings [28,29]. Chen et al. [28] demonstrated that the dominant eigenvalue of the covariance matrix (equivalent to the variance captured by the first principal component) increases as multivariate systems approach bifurcations, providing a “criticality index” that can detect resilience loss distributed across community members. Weinans et al. [29] systematically evaluated multivariate indicator performance, finding that composite indices integrating multiple variables generally outperform univariate approaches, particularly in high-dimensional systems where resilience loss may be heterogeneously distributed. Dynamic network biomarkers, originally developed for disease prediction [32,33], identify emergent modules of tightly correlated variables whose collective dynamics diverge from the rest of the system prior to transition; the framework has potential applicability to ecological networks where modular structure and changing inter-component correlations may presage regime shifts.

#### 5.2.2. Probabilistic and Bayesian Decision Frameworks

A fundamental shift in EWS methodology involves moving from deterministic alarms to probabilistic risk statements. Rather than asking whether “an early warning signal is present,” the operationally relevant question becomes the following: what is the probability of approaching a critical transition given the observed time series, and how does this probability change under alternative models and assumptions? This probabilistic perspective aligns more naturally with decision-making under uncertainty and provides a coherent framework for integrating multiple indicators with varying reliability [63]. It is also the natural framework in which to embed the decision-theoretic thresholds (acceptable false-alarm probabilities; expected loss functions) discussed in Section 5.4.

#### 5.2.3. Machine Learning and Deep Learning

Machine learning methods, particularly deep neural networks, have emerged as powerful tools for detecting early warning signals that may escape traditional statistical indicators. Bury et al. [30] developed EWSNet, a convolutional neural network trained on large ensembles of simulated time series approaching various bifurcation types; EWSNet learns combinations of features in univariate time series that indicate proximity to tipping points, achieving strong performance on simulated data and some empirical records. More recently, deep learning approaches have been extended to rate-induced tipping, a mechanism for which classical CSD-based indicators show limited utility, with Huang et al. [31] demonstrating that neural networks can detect fingerprints of approaching R-tipping events. The key challenges for machine-learning EWSs are transferability (performance may degrade when models trained on simulated data are applied to real ecological systems with different noise structures) and interpretability (the learned features may be difficult to connect to ecological mechanisms). Hybrid strategies in which machine-learning outputs serve as screening tools that prompt mechanistic follow-up are the most defensible path for operational adoption.

A complementary and rapidly developing strategy addresses the transferability problem directly by training ML models on empirical data rather than simulations. In data-rich domains, this approach has demonstrated that models can learn genuine pre-transition signatures from observed system behavior, avoiding the domain shift that degrades simulation-trained classifiers when applied to real time series. Hyland et al. [154] developed an empirically trained gradient-boosted ensemble to predict circulatory failure in intensive-care patients, training on over 54,000 hospital admissions and achieving early detection of 90% of events with substantially fewer false alarms than threshold-based monitors; crucially, they showed that performance degrades when the model is applied directly to an independent cohort without recalibration, underscoring that even empirically trained systems require local adaptation when the generating process shifts. In the financial domain, Samitas et al. [155] trained support-vector classifiers on empirical correlation and network-centrality features extracted from global equity, bond, and credit-default-swap markets, detecting contagion risk with over 98% accuracy on historical crises; they similarly found that predictive skill does not transfer across different network categories without retraining, reinforcing that data-rich empirical training yields high within-context performance but does not eliminate the need for system-specific calibration.

The ecological relevance of this empirical-training paradigm is illustrated most directly by Falmagne et al. [156], who constructed an EWS for regime shifts in a large-scale social system—the Reddit r/place collaborative canvas—exploiting the availability of thousands of comparable subsystems that each undergo abrupt transitions, providing a natural empirical training corpus unavailable in most ecological contexts. An XGBoost classifier trained on 175 features encoding seven hours of temporal memory—capturing image-activity dynamics, user coordination, attack-defence balance, and image complexity—detected 50% of transitions within 20 min with only 3.6% false positives (ROC AUC = 0.835) and remained predictive up to six hours in advance. Critically, a model trained on the 2022 event generalized meaningfully to the 2023 event despite substantial contextual differences, providing evidence that the classifier captures structural rather than incidental features of pre-transition dynamics. Interpretability via SHAP values revealed twelve distinct behavioral precursors, including signatures conceptually analogous to critical slowing down (elevated image instability) and critical speeding up (sustained defensive activity), alongside indicators of community discoordination and reduced image complexity. For ecological EWSs, the practical lesson from this body of work is twofold: where sufficiently dense observational records exist across multiple comparable subsystems—as is increasingly feasible with remote sensing and automated sensor networks—empirical training offers a route to ML classifiers that capture real pre-transition dynamics rather than idealized bifurcation scenarios; and the application of SHAP-based interpretability to such models provides a principled method for generating mechanistic hypotheses about precursory ecological processes that can then be evaluated against theory.

#### 5.2.4. Non-Equilibrium Thermodynamic Indicators

Classical EWS theory assumes systems near equilibrium, but many ecological systems operate under continuous forcing that maintains non-equilibrium dynamics. Xu et al. [34] applied landscape–flux theory to ecological models, demonstrating that quantities including average flux (the non-equilibrium driving force), entropy production rate (the thermodynamic cost of maintaining non-equilibrium), and time irreversibility (quantified through asymmetric cross-correlation functions) can serve as early warning signals. In model systems these indicators detected approaching transitions earlier than conventional CSD-based metrics, suggesting potential for improved lead times in practical applications. Their integration with classical EWSs is a promising frontier, though empirical validation in ecological data remains limited.

#### 5.2.5. Composite Indices and Multi-Indicator Synthesis

Given that individual EWSs can be noisy and sensitive to preprocessing choices, composite indicators that integrate multiple metrics computed on the same time series can substantially improve robustness. The goal extends beyond mere statistical aggregation to constructing indices with interpretable connections to resilience mechanisms and explicit performance evaluation under null models [63]. Composite approaches provide a natural interface for decision thresholds, allowing several weak temporal signals to be combined into stronger, calibrated risk scores suitable for management applications.

#### 5.2.6. Mechanism-Guided and Generalized Modelling

When partial knowledge of ecological processes is available, generalized modeling approaches can integrate structural information about feedback loops and interaction networks while remaining flexible about specific functional forms [157]. Such methods offer EWS diagnostics that are less dependent on long stationary time series, instead leveraging shorter records combined with mechanistic constraints, and connect more directly to stability theory and feedback structure than purely statistical patterns.

#### 5.2.7. State-Space and Geometric Indicators

Beyond moment-based statistics, the geometry of system trajectories in state space can encode resilience loss through changes in recurrence structure and attractor properties [158]. These geometric approaches complement traditional temporal indicators by capturing aspects of system behavior that may not be apparent in marginal statistics and provide a valuable conceptual expansion of what “early warning” can mean when systems are high-dimensional and subject to complex noise structures.

### 5.3. Complementary Role of Spatial Indicators

While time-series analysis remains the primary foundation of the EWS methodology, spatial dimensions provide an increasingly valuable complementary axis for early warning, particularly given the expanding availability of remote sensing and landscape-scale monitoring. In self-organized and patterned ecosystems, theory and empirical work demonstrate that shifts in spatial structure can precede desertification and other critical transitions [5,35]. Indicators such as increasing spatial variance, rising spatial autocorrelation, and changes in patch-size distributions may offer warnings even when long temporal records are unavailable.

A particularly promising frontier involves integrating temporal and spatial evidence. Remotely sensed products deliver dense spatial coverage, while local observatories provide higher-frequency temporal sampling and mechanistic depth. Methods that combine these information sources can yield more robust inference: spatial patterns may be less sensitive to certain forms of temporal measurement noise, while time-series indicators capture local recovery dynamics with greater precision. Recent work demonstrates the feasibility of using remote sensing to detect slowing down signals in spatially patterned dryland systems, highlighting the potential for multi-scale surveillance relevant to regional management [102].

### 5.4. Addressing Methodological Challenges

Despite the strong theoretical foundations and methodological advances described above, several challenges must be addressed to realize the full potential of time-series-based EWSs.

The most fundamental operational constraint is data availability. Many ecological time series lack the length, sampling frequency, or measurement quality required for reliable CSD-based detection. Irregular sampling, changes in measurement protocols, and short records reduce statistical power in ways that are difficult to compensate for analytically [19]. This limitation motivates a dual strategy: continued investment in long-term ecological observatories where feasible and parallel development of EWS methods that remain informative under suboptimal data conditions. The multivariate and mechanism-guided approaches reviewed in Section 5.2 represent partial responses to the second arm of this strategy, but the first— sustained institutional commitment to long-term monitoring—is ultimately a question of governance and funding that lies outside the reach of methodological innovation alone.

A second operational challenge concerns the translation of continuous indicator outputs into discrete management decisions. Policy and management frameworks typically require actionable categories (e.g., traffic-light risk levels or formal trigger thresholds), whereas EWS outputs are inherently continuous, probabilistic, and sensitive to analytical choices. Defining operationally meaningful thresholds—explicitly tied to management objectives such as acceptable false alarm probabilities or expected loss functions—is a practical prerequisite for institutional adoption that has received insufficient attention in the primary literature [63]. Probabilistic and Bayesian decision frameworks (Section 5.2.2) provide the most coherent conceptual basis for this translation, but ecosystem- specific calibration against empirical null distributions remains the norm rather than the exception.

A third challenge is institutional inertia and the gap between method development and practitioner uptake. The EWS toolkit has expanded rapidly, but accessible software pipelines with standardized workflows, transparent reporting norms, and preregistered detection protocols are only beginning to emerge [19]. Without these infrastructural elements, methodological advances risk remaining confined to the primary research literature rather than informing the monitoring programs and adaptive management frameworks where their impact would be greatest. User-oriented toolchains that deliver outputs as calibrated probabilistic risk summaries—rather than raw indicator time series—are an essential next step toward bridging this gap.

### 5.5. Emerging Applications and Cross-System Synthesis

The conceptual and methodological framework of EWSs, developed primarily through analysis of ecological time series, is increasingly being applied beyond canonical ecosystem regime shifts. In coupled social–ecological systems, feedbacks between human behavior and ecological state can produce abrupt changes in fisheries, agriculture, and resource governance. Comparative synthesis of regime shifts across systems highlights the diversity of drivers and the relevance of cross-scale interactions, underscoring the need for monitoring frameworks that track both ecological state variables and human pressures through integrated temporal data streams [159].

At planetary scales, EWS methodologies are being explored for climate and Earth-system tipping elements, including large-scale circulation patterns and biome transitions. Recent synthesis work connects methodological developments across climate, ecological, and human systems, emphasizing both opportunities and challenges in interpreting early warnings under complex, nonstationary forcing [41]. The extension of ecological EWS methods to biomedical contexts—including microbiome stability and dysbiosis—suggests that resilience diagnostics based on temporal dynamics may become a broadly unifying framework across natural and human systems.

### 5.6. Priority Directions for Future Research

Several research directions are especially consequential for advancing EWS science and practice in the coming years.

Prospective and real-time validation represents perhaps the most critical need. The majority of published EWS applications remain retrospective, analyzing historical transitions with the benefit of hindsight. Prospective tests in long-term observatories and controlled field experiments are essential to quantify predictive skill, false alarm rates, and practical lead times under realistic operational conditions [19,74].

Multi-scale data integration offers substantial promise for improving EWS robustness. Combining fine-scale field monitoring, remote sensing, and Earth-system modeling can enable surveillance across spatial and temporal scales relevant for management. This requires methodological advances in data fusion, scale alignment, and uncertainty propagation that preserve the strengths of each data source [41,102].

User-oriented toolchains and transparent reporting will determine whether methodological advances translate into widespread adoption. Accessible software pipelines, standardized analytical workflows, and reporting norms (including sensitivity analyses, null-model benchmarking, and ideally preregistered detection protocols) are needed. Delivering outputs as probabilistic risk levels with interpretable summaries will be essential for management uptake.

Finally, hybrid frameworks accommodating diverse tipping mechanisms represent the most reliable pathway to actionable early warning. Given the limitations of CSD-centric indicators for certain transition types, methods that blend mechanistic modeling with statistical and machine learning approaches, while continuously tracking stressor trajectories, will likely prove most effective in systems subject to shocks and rapid environmental forcing [20,30,157].

These operational and inferential challenges have been brought into sharp focus by recent empirical reassessments. O’Brien et al. [19] conducted a comprehensive analysis of EWSs in long-term lake monitoring data, finding that traditional CSD-based indicators showed limited and inconsistent ability to anticipate documented transitions; their analysis highlighted the gap between theoretical expectations and empirical performance, emphasizing the challenges posed by short time series, environmental noise, and transitions that do not conform to classical bifurcation models. The Global Tipping Points Report [160] synthesized EWS research across climate, ecological, and human systems, concluding that while EWSs show promise for systems with well-characterized bifurcation dynamics, their operational utility for prospective prediction remains to be demonstrated in most contexts. These reassessments do not invalidate the EWS approach but clarify its scope of applicability: EWSs are most reliable when applied to systems whose dynamics are reasonably approximated by low-dimensional models approaching fold-type bifurcations, with sufficiently long and high-quality time series, and where alternative tipping mechanisms (N-, R-, S-tipping) can be reasonably excluded.

## 6. Conclusions

Early warning signal research in ecology has evolved from a theoretical endeavor grounded in dynamical systems theory to an empirical and methodological enterprise with applications across diverse ecosystem types. The present review complements existing syntheses—which span Earth-system, ecological, and human contexts at high generality [37,38,39,41], by restricting its scope to ecological systems, integrating explicit methodological guidance on the analytical pipeline (windowing, detrending, trend detection, and sensitivity analysis), and organizing the ecosystem-by-ecosystem evidence around the B/N/R/S typology of tipping mechanisms developed in Section 2.6. These three emphases are the principal contributions of this review.

A unifying thread of the synthesis is that the applicability of CSD-based indicators is not a property of EWS methodology in the abstract, but of the specific tipping mechanism operating in each ecosystem. The B/N/R/S classification developed in Section 2.6, summarized in Table 3, and applied in each ecosystem subsection makes this dependence explicit: shallow lakes approach eutrophication primarily through B-tipping and yield the strongest empirical evidence for classical CSD signatures; coral reefs are dominated by S-tipping and yield weak and inconsistent classical EWSs, with flickering and bistability precursors providing more informative signals; grasslands and drylands combine B-tipping (vegetation–soil feedbacks) with R-tipping under rapid climate forcing and yield more reliable spatial than temporal indicators; marine fisheries and pelagic systems span the full B/N/R/S range case-by-case; and forest systems exhibit B-tipping at the landscape scale and R-/S-tipping at the stand scale, with CSD detected primarily through satellite-derived recovery rates rather than stand-level demography. Read through this typological lens, the apparent heterogeneity of empirical EWS performance is not a failure of the framework but a direct consequence of mechanistic heterogeneity, and the practical question shifts from “do EWSs work?” to “which indicators are theoretically expected to work in this system, and have the relevant confounders been controlled?”. Distinguishing tipping mechanisms from observational data is itself a nontrivial inferential problem [110], which reinforces rather than weakens the case for pre-classifying systems before deploying EWSs.

Several cross-cutting findings follow directly from this organization. First, no single indicator is universally reliable; the statistical power of variance, autocorrelation, skewness, and other metrics (Table 1) varies substantially with data quality, transition mechanism, and system-specific dynamics, motivating the composite indices and multi-indicator frameworks discussed in Section 5.2. Second, the qualitative direction of the expected indicator trend is itself not universal: critical speeding up (CSU) [69,71] produces the opposite signatures from CSD under certain geometries of parameter change, and standard one-sided trend tests are insufficient when the sign of the expected trend cannot be predicted a priori. Third, spatial and temporal indicators provide complementary information, with spatial metrics often offering greater sensitivity in patterned ecosystems and enabling resilience assessment from landscape snapshots when long time series are unavailable [35,36,161].

Realizing the practical potential of these methods will require progress on the operational and institutional fronts identified in Section 5: prospective validation in real monitoring contexts, multi-scale data integration, accessible toolchains, and hybrid frameworks that accommodate the full diversity of tipping mechanisms encountered in practice. The retrospective character of the bulk of the empirical literature reviewed here is the single most consequential limitation of the current evidence base, and addressing it will require the kind of pre-registered, prospectively designed monitoring programs that have begun to appear in adjacent fields but remain rare in ecology.

Time-series-based early warning signals are neither infallible oracles nor theoretical curiosities. They are diagnostic tools whose utility depends on appropriate application, transparent reporting of uncertainties, integration with mechanistic understanding, and embedding within broader frameworks of ecological monitoring and adaptive management. The B/N/R/S typology, the second-generation methodological toolkit, and the consolidating empirical literature reviewed here collectively suggest that the field is approaching a mature operational form—one in which the question is no longer whether CSD-based indicators detect approaching transitions, but when, for which mechanisms, and under what inferential safeguards. The combination of this mechanistic clarification with continued methodological development and the overdue institutional commitment to prospective testing offers, in our view, the most promising pathway toward an anticipatory ecology adequate to the pressures of the Anthropocene.

## Figures and Tables

**Table 1:** Common statistical indicators as early warning signals (EWSs) based on time-series analysis. Each indicator relates to theoretical changes expected when approaching a tipping point, assuming primarily internal variability (white noise forcing) and proximity to a catastrophic bifurcation exhibiting critical slowing down (CSD).

EWS Indicator	Mathematical Definition	Property Measured	Expected Change Before Transition	Limitations/Caveats
**Lag-1 Autocorrelation (AR(1))**	$\rho_{1}=\frac{\sum_{t=1}^{n-1}\left(x_{t}-\bar{x}\right)\left(x_{t+1}-\bar{x}\right)}{\sum_{t=1}^{n}{\left(x_{t}-\bar{x}\right)}^{2}}$	Temporal memory at lag-1. Inversely related to the return rate: $\rho_{1}\approx e^{\lambda \Delta t}$.	**Increases** toward 1 as the bifurcation approaches, reflecting critical slowing down [37,38].	Sensitive to detrending method; false positives under non-stationary forcing; assumes linear dynamics near equilibrium.
**Variance**	$\sigma^{2}=\frac{1}{n-1}\sum_{t=1}^{n}{\left(x_{t}-\bar{x}\right)}^{2}$	Amplitude of fluctuations (2nd central moment). Often reported as σ or $CV=\sigma /|\bar{x}|$.	**Increases**. Reduced stability amplifies perturbations; flickering also elevates variance before collapse [72,74].	Confounded by changes in forcing magnitude; sensitive to outliers and noise non-stationarity.
**Skewness**	$\gamma_{1}=\frac{\frac{1}{n}\sum_{t=1}^{n}{\left(x_{t}-\bar{x}\right)}^{3}}{\sigma^{3}}$	Asymmetry of the distribution (3rd standardized moment).	**Typically increases**. Distribution becomes skewed toward the alternative regime. Flickering generates asymmetric tails. Sign depends on transition direction [72,78].	Highly sensitive to outliers; sign interpretation requires knowledge of system geometry; needs large samples.
**Kurtosis**	$\gamma_{2}=\frac{\frac{1}{n}\sum_{t=1}^{n}{\left(x_{t}-\bar{x}\right)}^{4}}{\sigma^{4}}-3$	Tail heaviness (4th standardized moment). Measures frequency of extreme values vs. Gaussian.	**Increases**. Extreme fluctuations become more frequent; flickering produces heavy tails from stochastic jumps between attractors [72,78].	Extremely sensitive to outliers; requires very large samples; 4th moment has high variance.
**Power Spectrum (Spectral Reddening)**	$S\left(f\right)\propto f^{-\beta }$ Spectral exponent β from log-log slope.	Variance distribution across frequencies. Summarized by exponent β or low/high frequency power ratio.	**Shifts toward low frequencies**. Slow fluctuations dominate; spectrum reddens with β→−1 (flicker noise) [16,79,87].	Requires long, evenly sampled series; confounded by trends; spectral leakage bias.
**Entropy Indicators**	Permutation: $H_{p}=-\sum_{i=1}^{m!}p_{i}\ln p_{i}$ Shannon: $H_{S}=-\sum_{i=1}^{k}p_{i}\ln p_{i}$	Complexity and regularity. Low entropy = predictable dynamics; high entropy = random behavior.	**Typically decreases**. More correlated dynamics reduce ordinal pattern diversity, indicating fewer accessible states and loss of resilience [27].	Depends on embedding parameters; may increase in some systems; interpretation is system-specific.
**Detrended Fluctuation Analysis (DFA)**	$F\left(s\right)\propto s^{\alpha }$α: DFA exponent (α=0.5: white noise; α=1: critical).	Long-range correlations; scaling of fluctuations across time scales. Related to Hurst exponent.	**Increases** toward 1.0, indicating memory across multiple temporal scales typical of critical dynamics [38,80].	Requires very long series; sensitive to non-stationarities and detrending order; crossover effects complicate interpretation.
**Return Rate**	$\lambda =-\frac{\ln(\rho_{1})}{\Delta t}$ Or $\lambda =1/\tau_{\mathrm{rec}}$ from recovery time.	Rate of return to equilibrium after perturbation. Direct measure of dominant eigenvalue.	**Decreases** toward zero—the direct manifestation of critical slowing down [13,60].	Requires known sampling interval; assumes linear dynamics; experimental measurement needs controlled perturbations.
**Conditional Heteroskedasticity**	GARCH(1,1): $\sigma_{t}^{2}=\omega +\alpha_{1}\epsilon_{t-1}^{2}+\beta_{1}\sigma_{t-1}^{2}$	Time-varying volatility; measures whether variance clusters in time (volatility persistence).	**Increases**. Variance becomes more dependent on recent fluctuations; $\alpha_{1}+\beta_{1}\to 1$ indicates persistent volatility [88].	Requires long series; model selection is non-trivial; assumes parametric volatility form.
**Potential Analysis (Bimodality)**	$BC=\frac{\gamma_{1}^{2}+1}{\gamma_{2}+3}$BC>0.56 suggests bimodality.	Shape of stability landscape; presence of alternative stable states.	**Bimodality increases**. Potential barrier between states decreases; distribution develops two modes [89].	BC is a rough heuristic; potential reconstruction assumes quasi-static equilibrium; sensitive to bandwidth.
**Cross-Correlation**	$\rho_{xy}=\frac{\mathrm{Cov}(x,y)}{\sigma_{x}\sigma_{y}}$	Linear association between system variables; synchronization and coupling strength.	**Increases**. Components become more coupled as all variables slow down together and respond coherently [38].	Requires multiple variables; sensitive to common drivers; does not distinguish direct from indirect coupling.
**Dynamic Network Biomarkers (DNB)**	$I_{\mathrm{DNB}}=\frac{\bar{PCC_{d}}\cdot \bar{SD_{d}}}{\bar{PCC_{o}}}$ where $PCC_{d}$ denotes intra-module correlation, $SD_{d}$ the standard deviation of the dominant module, and $PCC_{o}$ the correlation with other modules.	Detects modules of highly correlated variables whose collective dynamics diverge from the remainder of the system prior to transition.	**Increases abruptly**. A subset of variables becomes highly correlated internally, exhibiting elevated variance and decoupling from the rest of the system [32,90,91].	Requires high-dimensional data (omics, networks); identification of the dominant module may be ambiguous; assumes modular system structure.
**Criticality Index**	$CI=\frac{\lambda_{1}}{\sum_{i=1}^{n}\lambda_{i}}$ where $\lambda_{1}$ is the largest eigenvalue of the covariance matrix and $\sum \lambda_{i}$ the total variance.	Fraction of variance explained by the first principal component; measures dominance of a single collective mode.	**Increases** toward 1. System dynamics become dominated by a single collective mode, indicating loss of effective dimensionality [92,93].	Sensitive to the number of variables; requires multivariate data; may be confounded by common external forcing.
**Fisher Information**	$FI=\int \frac{1}{p(x)}{\left(\frac{\partial p(x)}{\partial \theta }\right)}^{2}dx$ Empirical estimation via changes in probability distribution.	Sensitivity of the system to changes in control parameters; quantifies the degree of order within the system.	**Decreases**. Reduced capacity of the system to distinguish between states; loss of order and increased uncertainty prior to collapse [25,26].	Empirical estimation requires discretization sensitive to bin selection; interpretation depends on the choice of control parameter.
**Network Correlation**	$\bar{\rho }=\frac{2}{n(n-1)}\sum_{i<j}\rho_{ij}$ Mean pairwise correlation among *n* system variables.	Global synchronization; average degree of coupling among system components.	**Increases**. All components respond more coherently to forcing due to generalized critical slowing down [38,94].	Does not distinguish direct from indirect correlation; sensitive to common drivers; requires multiple simultaneous time series.
**Hysteresis**	$H=|x_{\mathrm{forward}}\left(\theta \right)-x_{\mathrm{backward}}\left(\theta \right)|$ Difference between forward and backward trajectories in parameter space.	Irreversibility; path-dependence of system state. Indicates the presence of alternative stable states.	**Emerges or increases**. The system exhibits different transition thresholds depending on the direction of parameter change, confirming bistability [1,43].	Requires experimental manipulation of the control parameter in both directions; difficult to detect in observational systems; requisite time scales may be prohibitive.

**Table 2:** Summary of EWSs reported across different ecosystem types and critical transitions.

Ecosystem (Transition)	Observed Early Warning Signals	Key References
Shallow Lake (Eutrophication)	Increasing lag-1 autocorrelation (AR(1)) and standard deviation in water quality parameters; flickering dynamics (oscillations between clear and turbid states) observed years before definitive regime shift; bimodal distribution of nutrient indicators preceding final collapse.	Wang et al. [81]
Experimental Lake (Trophic Cascade)	Progressive increases in autocorrelation and variance of phytoplankton density; decreased return rate from small perturbations measured in situ (critical slowing down); increased skewness in water transparency distribution prior to transition.	Carpenter et al. [101]
Semiarid Grassland (Desertification)	Increased spatial variance in NDVI; vegetation patch patterns becoming more connected (indicating spatial synchronization); rising temporal autocorrelation in productivity indices; reduced resilience manifested as slower recovery following drought events.	Kéfi et al. [5]; Veldhuis et al. [102]
African Savanna (Herbivore Collapse)	Increased interannual variance in population counts; shifts in age structure (reduced proportion of juveniles); elevated correlation among population dynamics of different herbivore species (synchronized decline across taxa).	Dakos et al. [50]
Coral Reef (Algal Dominance)	Elevated temporal persistence (AR(1)) in coral cover monitoring data; increasing interannual variance in macroalgal density; sporadic episodes of transient algal dominance (flickering) before permanent regime establishment; decline in herbivorous fish diversity.	Mumby et al. [47]; Dakos et al. [63]
Tropical Forest (Amazon Savannization)	Rising autocorrelation and variance in NDVI, *Vegetation Optical Depth (VOD)*, and evapotranspiration time-series; delayed recovery of vegetation greenness following droughts (critical slowing down detected via satellite); increased synchronization of fire activity across large areas; spatial flickering dynamics.	Verbesselt et al. [103]; Boulton et al. [104]
Boreal Forest (Post-Fire Collapse)	Repeated observations of reduced seedling density following fire events (declining resilience); increased variance among plots in regeneration rates; rising temporal autocorrelation in annual vegetation greenness indices prior to mass mortality events.	Carpenter and Brock [74]; Scheffer et al. [105]
Marine Fishery (Stock Collapse)	Increased interannual variability in recruitment; elevated autocorrelation in catch and biomass time-series; reduced resilience indices; demographic signals including decreasing proportion of young individuals years before collapse.	Biggs et al. [106]; Litzow et al. [107]
Pelagic Ocean (Plankton Regime Shift)	Spectral reddening of climate–biological variability (increased low-frequency power); rising variance in plankton indices in preceding decades; synchronization of previously anti-phase population oscillations before species collapse.	Hsieh et al. [108]; Conversi et al. [109]

AR(1) = lag-1 autocorrelation coefficient; NDVI = Normalized Difference Vegetation Index. Flickering refers to transient oscillations between alternative states prior to permanent transition.

**Table 3:** Dominant tipping mechanisms, expected applicability of CSD-based early warning signals, and principal confounders by ecosystem class. Mechanism codes follow Section 2.6: B = bifurcation-induced, N = noise-induced, R = rate-induced, and S = shock-induced. Applicability is a theoretical expectation under the assumption that the listed mechanism dominates; empirical performance is reviewed in the corresponding subsection. The dagger (^†^) marks ecosystems in which the canonical CSD signature may invert into critical speeding up (CSU) under Allee-driven dynamics; see Section 2.3 and note below.

Ecosystem (Section)	Dominant Mechanism (s)	Expected CSD Applicability	Principal Confounders
Shallow lakes—eutrophication (Section 4.2)	B (dominant); N, S (secondary)	High for slow nutrient loading; degraded by flickering and pulse loading	Seasonality; short records; non-stationary nutrient inputs; sensor changes
Coral reefs (Section 4.3)	S (dominant); B, N (secondary)	Low to moderate; classical CSD often absent before bleaching shocks	Acute thermal events; storm damage; disease outbreaks; spatial heterogeneity
Grasslands and drylands (Section 4.1)	B (vegetation–soil feedbacks); R (rapid climate forcing)	Moderate; spatial EWSs often more reliable than temporal	Non-stationary precipitation; land-use change; rising forcing variance (σ-inflation)
Marine fisheries and pelagic systems ^†^ (Section 4.4)	Full B/N/R/S range across cases	Heterogeneous; case-by-case (see Section 4.4)	Fishing inflates variance independently of $\lambda_{1}$ [108]; ENSO/PDO forcing; model-derived stock data; Allee dynamics may produce CSU rather than CSD [71]
Forests—Amazon, boreal ^†^ (Section 4.5)	B (vegetation–climate feedbacks); R (rapid drying); S (fire, drought pulses)	Moderate at landscape scale via remote sensing; weak at stand scale	Long demographic timescales; lagged responses; fire–vegetation coupling; potential CSU under Allee-driven recruitment failure

^†^ In fold-bifurcation systems whose control parameter compresses rather than flattens the basin of attraction—notably Allee-driven population dynamics relevant to depensatory recruitment in exploited fish stocks (Section 4.4) and to recruitment collapse in slow-regenerating tree populations (Section 4.5)—classical CSD signatures may invert into critical speeding up (CSU), with variance and autocorrelation decreasing rather than increasing as the transition is approached [69,71]. Practitioners working with these systems should consider two-sided trend tests or model-informed sign predictions when interpreting EWS indicators; see Section 2.3 and Section 3.1.3.

## Data Availability

No data were used for the research described in this review.

## Abbreviations

The following abbreviations are used in this manuscript:

EWS	Early Warning Signal
CSD	Critical Slowing Down
AR(1)	Lag-1 Autocorrelation
